# Major role of iron uptake systems in the intrinsic extra-intestinal virulence of the genus *Escherichia* revealed by a genome-wide association study

**DOI:** 10.1371/journal.pgen.1009065

**Published:** 2020-10-28

**Authors:** Marco Galardini, Olivier Clermont, Alexandra Baron, Bede Busby, Sara Dion, Sören Schubert, Pedro Beltrao, Erick Denamur

**Affiliations:** 1 EMBL-EBI, Wellcome Genome Campus, Cambridge, United Kingdom; 2 Université de Paris, IAME, UMR1137, INSERM, Paris, France; 3 Genome Biology Unit, EMBL, Heidelberg, Germany; 4 Max von Pettenkofer Institute of Hygiene and Medical Microbiology, Faculty of Medicine, LMU Munich, Germany; 5 AP-HP, Laboratoire de Génétique Moléculaire, Hôpital Bichat, Paris, France; University of Warwick, UNITED KINGDOM

## Abstract

The genus *Escherichia* is composed of several species and cryptic clades, including *E*. *coli*, which behaves as a vertebrate gut commensal, but also as an opportunistic pathogen involved in both diarrheic and extra-intestinal diseases. To characterize the genetic determinants of extra-intestinal virulence within the genus, we carried out an unbiased genome-wide association study (GWAS) on 370 commensal, pathogenic and environmental strains representative of the *Escherichia* genus phylogenetic diversity and including *E*. *albertii* (n = 7), *E*. *fergusonii* (n = 5), *Escherichia* clades (n = 32) and *E*. *coli* (n = 326), tested in a mouse model of sepsis. We found that the presence of the high-pathogenicity island (HPI), a ~35 kbp gene island encoding the yersiniabactin siderophore, is highly associated with death in mice, surpassing other associated genetic factors also related to iron uptake, such as the aerobactin and the *sitABCD* operons. We confirmed the association *in vivo* by deleting key genes of the HPI in *E*. *coli* strains in two phylogenetic backgrounds. We then searched for correlations between virulence, iron capture systems and *in vitro* growth in a subset of *E*. *coli* strains (N = 186) previously phenotyped across growth conditions, including antibiotics and other chemical and physical stressors. We found that virulence and iron capture systems are positively correlated with growth in the presence of numerous antibiotics, probably due to co-selection of virulence and resistance. We also found negative correlations between virulence, iron uptake systems and growth in the presence of specific antibiotics (*i*.*e*. cefsulodin and tobramycin), which hints at potential “collateral sensitivities” associated with intrinsic virulence. This study points to the major role of iron capture systems in the extra-intestinal virulence of the genus *Escherichia*.

## Author summary

Bacterial isolates belonging to the genus *Escherichia* can be human commensals but also opportunistic pathogens, with the ability to cause extra-intestinal infection. There is therefore the need to identify the genetic elements that favour extra-intestinal virulence, so that virulent bacterial isolates can be identified through genome analysis and potential treatment strategies be developed. To reduce the influence of host variability on virulence, we have used a mouse model of sepsis to characterize the virulence of 370 strains belonging to the genus *Escherichia*, for which whole genome sequences were also available. We have used a statistical approach called Genome-Wide Association Study (GWAS) to show how the presence of genes that encode for iron scavenging are significantly associated with the propensity of a bacterial isolate to cause extra-intestinal infections. Taking advantage of previously generated growth data on a subset of the strains and its correlation to virulence we generated hypothesis on the relationship between iron scavenging and growth in the presence of various antimicrobials, which could have implications for developing new treatment strategies.

## Introduction

Members of the *Escherichia* genus are both commensals of vertebrates [1] and opportunistic pathogens [2] involved in a wide range of intestinal and extra-intestinal infections. Apart from the *E*. *coli* species, the genus is composed of the cryptic *Escherichia* clades, and the *E*. *fergusonii* and *E*. *albertii* species. The latter taxa are rarely isolated in humans but are more frequently found in the environment and avian species where they can cause intestinal infections [3–5]. In humans, extra-intestinal infections represent a considerable burden [6], with bloodstream infections (bacteraemia) being the most severe with a high attributable mortality of between 10–30% [7–10]. The regular increase over the last 20 years of *E*. *coli* bloodstream incidence [11] and antibiotic resistance [12] is particularly worrisome. The factors associated with high mortality are mainly linked to host conditions such as age, the presence of underlying diseases and to the portal of entry, with the urinary origin being more protective. These factors outweigh those directly attributable to the bacterial agent [7–9,13].

Nevertheless, the use of animal models has shown a great variability in the intrinsic extra-intestinal virulence potential of natural *Escherichia* isolates. In a mouse model of sepsis where bacteria are inoculated subcutaneously, it has been clearly shown that the intrinsic virulence quantified by the number of animal deaths over the number of inoculated animals for a given strain is dependant on the number of virulence factors such as adhesins, toxins, protectins and iron capture systems [14–19]. One of the most relevant virulence factors is the so-called high-pathogenicity island (HPI), a 36 to 43 kb region encoding the siderophore yersiniabactin, a major bacterial iron uptake system [20], which has also been shown to reduce the efficacy of innate immune cells to cause oxidative stress [21]. The deletion of the HPI results in a decrease in the intrinsic virulence in the mouse model in a strain-dependent manner [16,18,22], indicating complex interactions between the genetic background of each strain and the HPI.

The limitation of these gene inactivation studies is that they target specific candidate genes and cannot be performed in a large number of strains. Recently, the development of new approaches in bacterial genome-wide association studies (GWAS) [23–26] allows searching in an unbiased manner for genotypes associated with specific phenotypes such as drug resistance or virulence in numerous strains. In this context, we conducted a GWAS in 370 commensal and pathogenic strains of *E*. *coli*, and related *Escherichia* clades, as well as *E*. *fergusonii* and *E*. *albertii*, representing the genus phylogenetic diversity, to search for traits associated with virulence in the mouse model of sepsis [27]. Most of the strains were isolated from a human host and are divided between commensals and extra-intestinal pathogens. Most importantly, many (N = 186) of these strains have been recently phenotyped across hundreds of growth conditions, including antibiotics and other chemical and physical stressors [28]. This data could then be used to find phenotype associations with virulence and to generate hypotheses on the function of genetic variants associated with the extra-intestinal virulence phenotype and their role for growth in those conditions.

## Results

### GWAS identifies the high-pathogenicity island as the strongest driver of the extra-intestinal virulence phenotype

**Fig 1:**
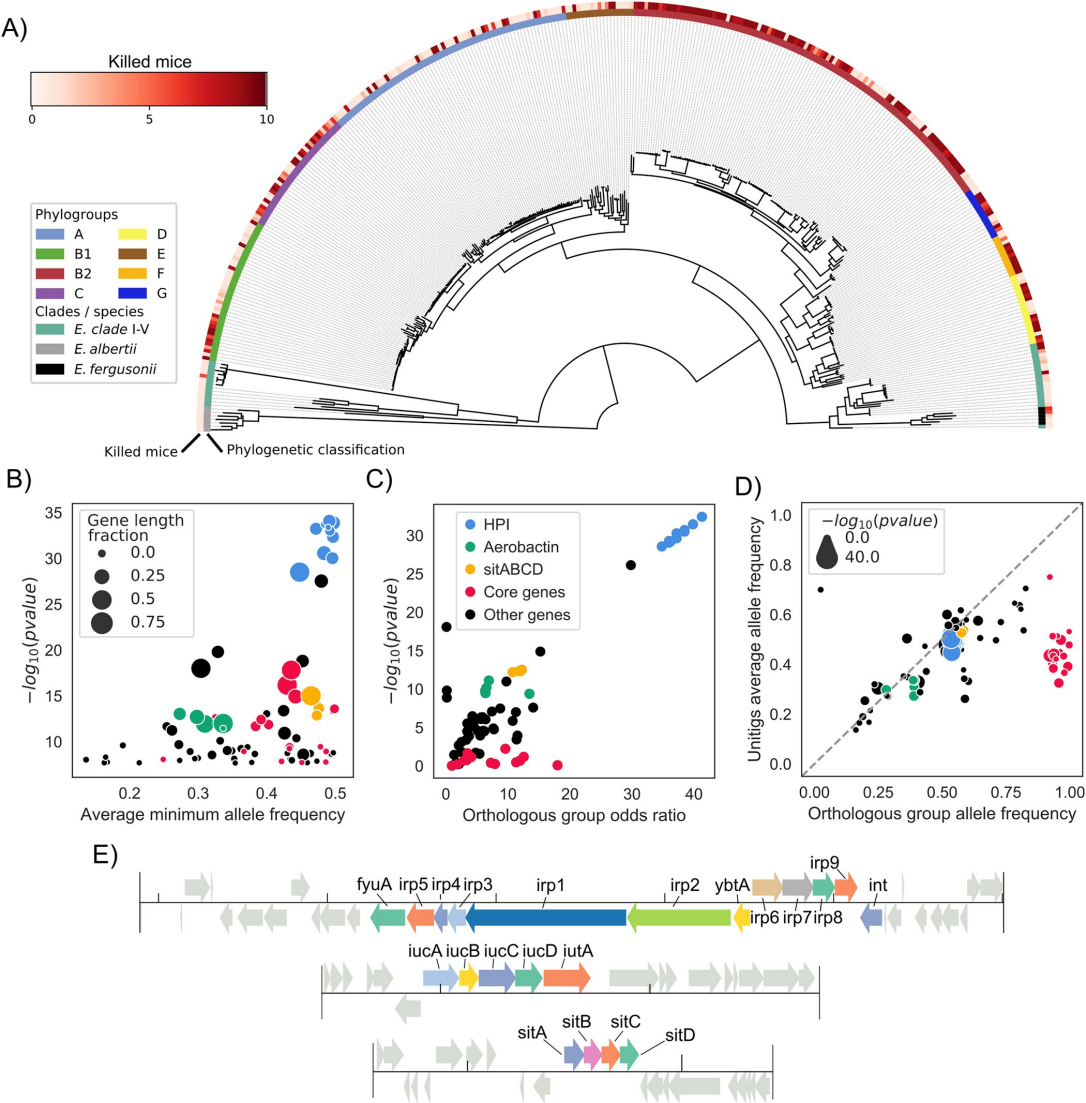
The HPI is strongly associated with the extra-intestinal virulence phenotype assessed in the mouse sepsis assay. A) Core genome phylogenetic tree of the *Escherichia* strains used in this study rooted on *E*. *albertii* strains. Outer ring reports virulence as the number of killed mice over the 10 inoculated per strain, inner ring the phylogroup, clade or species each strain belongs to. B) Results of the unitigs association analysis: for each gene the minimum association p-value and average minimum allele frequency (MAF) across all mapped unitigs is reported. The gene length fraction is computed by dividing the total length of mapped unitigs by the length of the gene. The color of each gene follows the same key as panel C. C) Results of the gene presence/absence association analysis; only those genes with at least one associated unitig mapped to them are represented. D) Scatterplot of gene frequency versus frequency of associated unitigs; points on the diagonal indicate hits where the association is most likely due to a gene’s presence/absence pattern rather than a SNP. The color of each gene follows the same key as panel C. E) The structure of the HPI and of the aerobactin and *sitABCD* operons in strain IAI39; all associated genes are highlighted.

We studied a 326 strain collection representative of the *E*. *coli* phylogenetic diversity, with strains belonging to phylogroups A (N = 72), B1 (N = 41), B2 (N = 111), C (N = 36), D (N = 20), E (N = 19), F (N = 12) and G (N = 15). To have a broader phylogenetic representation, which could increase statistical power [24,29], we also included strains from *Escherichia* clades I to V (N = 32) and the species *E*. *albertii* (N = 7) and *E*. *fergusonii* (N = 5) [30]. These strains encompass 170 commensal strains and 187 strains isolated in various extra-intestinal infections, mainly urinary tract infections and bacteraemia [7,14,31–37]. The isolation host is predominantly humans (N = 291), followed by animals (N = 72) and isolates from environmental sources (N = 6). To avoid any bias linked to host conditions, we assessed the strain virulence as its intrinsic extra-intestinal pathogenic potential using a well-calibrated mouse model of sepsis [14,27], expressed as the number of killed mice over the 10 inoculated per strain. In accordance with previous data [14,17,27,38,39], phylogroup B2 is the most associated with the virulence phenotype (2E^-9^ Wald test p-value, Fig 1A, S1 Table).

We used a bacterial GWAS method to associate unitigs—which are nodes in a colored de Bruijn graph representing a contiguous DNA sequence shared by one or more samples—to the virulence phenotype, allowing us to simultaneously test the contribution of core and accessory genome variation to pathogenicity [25]. It is generally understood that such methods require large sample sizes and phylogenetic diversity to have sufficient power, due to the need to observe multiple independent acquisitions of causal variants across clades and distinguish them from lineage defining variants; the appropriate sample size is also a function of the penetrance of the causal variants [24,29]. We ran simulations with an unrelated set of complete *E*. *coli* genomes and verified that our sample size was appropriate for variants with high penetrance and intermediate frequency (i.e. odds ratio above 5 and minor allele frequency > 0.1, S1 Fig, Methods). We reasoned that some of the genetic determinants of virulence are likely to have a relatively high penetrance due to the selective advantage they might confer in opening up a new niche [40,41], and that the strains used were phylogenetically diverse, enough to reach sufficient statistical power.

We uncovered a statistically significant association between 5,214 unitigs and the virulence phenotype, which were mapped back to 81 genes across the strains’ pangenome (Fig 1B, S2 Table, Methods). We carried out a gene ontology (GO) term enrichment analysis on the 81 genes, and found that 7 terms were significantly enriched (FDR-corrected p-value < 0.05, S3 Table); among those 6 were related to iron homeostasis (such as GO:0030091, “response to iron ion”), and one to protein repair (GO:0030091). To understand whether the presence of these 81 genes is directly associated with virulence or if it is due to genetic variants such as SNPs we performed a separate association analysis using genes’ presence absence patterns. This showed that most genes have an odds ratio that far exceeds the required threshold we estimated from simulations, as well as low association p-value (Fig 1C). Furthermore, 48 out of 81 genes with at least one associated unitig mapped to them have a frequency across strains that is highly correlated with that of the associated unitigs (Fig 1D), indicating that it’s the presence/absence pattern of those genes to be associated with virulence and not other kinds of genetic variants such as SNPs mapping to those genes.

Genes belonging to the HPI had the lowest association p-value by far (<1E^-28^); the presence of genes belonging to two additional operons encoding for bacterial siderophores (aerobactin [42] and *sitABCD* [43]) was also found to be associated with virulence (Fig 1E). We found that the HPI structure was highly conserved across the genomes that encode it (S2 Fig). We also observed that the distribution of a collection of some known virulence factors [44] didn’t match the virulence phenotype as closely as the HPI or the aerobactin and *sitABCD* operons, or had unitigs passing the association threshold (p-value > 2.16E^-08^, gene presence/absence patterns shown in S4 Fig), suggesting how iron scavenging is an important factor in determining virulence.

Among the remaining 33 genes with associated unitigs out of 81 total, 18 have a high frequency in the pangenome (> 0.9) and a low gene length fraction (i.e. the associated unitigs cover only a fraction of the gene, < 50%, Fig 1B), indicating that the presence of genetic variants such as SNPs present in core genes is associated to the virulence phenotype. We found that the core genes with the lowest association p-values were: *zinT* (p-value 1E^-16^), encoding a zinc and cadmium binding protein [45], *mtfA* (p-value 1E^-14^), encoding a protein involved in the regulation of carbohydrate metabolism [46], *shiA* (p-value 1E^-14^), encoding a transporter of shikimate, a compound involved in siderophore synthesis [47,48], *hprR* and *hprS* (p-value 1E^-13^ and 1E^-9^, respectively), encoding a two-component regulatory systems involved in the response to hydrogen peroxide [49] and *msrPQ* (p-value 1E^-12^ for both genes) an operon encoding enzymes involved in repairing periplasmic proteins under oxidative stress [50]. Most of these core genome hits (14 over 18 total) are encoded in the region surrounding the HPI (S3 Fig), which might imply that these hits are correlated with the presence of the HPI and not causally linked with extra-intestinal virulence. The remaining four core genome hits include *rspB* (p-value 1E^-8^), encoding a starvation sensing protein, and *torD* (p-value 1E^-8^), part of the *torCAD* operon involved in anaerobic respiration with trimethylamine-N-oxide (TMAO) as an electron acceptor [51,52].

### Gene knockout experiments validate the role of the HPI in the extra-intestinal phenotype

**Fig 2:**
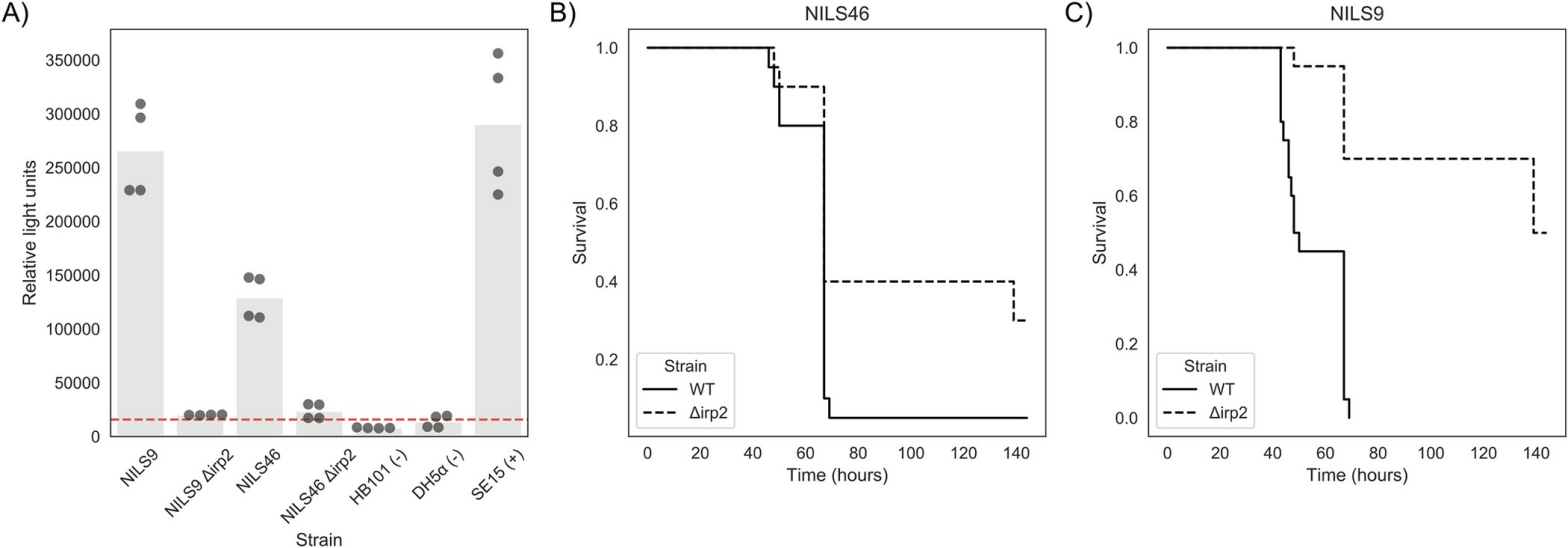
Phenotypic consequences of HPI deletion. A) Deletion of HPI leads to a decrease in production of yersiniabactin. Production of yersiniabactin is measured using a luciferase-based reporter (Methods). Strains marked with a “-” and “+” sign indicate a negative and positive control, respectively. The red dashed line indicates an arbitrary threshold for yersiniabactin production, derived from the average signal recorded from the negative controls plus two standard deviations. B-C) Deletion of HPI leads to an increase in survival after infection. Survival curves for wild-type strains and the corresponding *irp2* deletion mutant, built after infection of 20 mice for each strain. B) Survival curve for strain NILS46. C) Survival curve for strain NILS9.

Previous studies on the role of the HPI in experimental virulence gave contrasting results according to the strains’ genetic background [18]. Among B2 phylogroup strains, HPI deletion in the 536 strain (ST127; ST: sequence type) did not have any effect in the mouse model of sepsis [53] whereas this deletion in the NU14 strain (ST95) dramatically attenuated virulence [18]. Two strains from the present study belonging to B2 phylogroup/ST141 (IAI51 and IAI52) deleted in the longest gene of the HPI (*irp1)* have attenuated virulence in the same mouse model [22]. Deletion of the second longest gene of the HPI (*irp2*) in a strain (A1749) belonging to phylogroup D (ST69) also showed attenuated virulence in the same sepsis model [54]. We further documented the role of the HPI in extraintestinal virulence constructing *irp2* deletion gene mutants in two additional strains of phylogroup D (NILS46, ST69) and A (NILS9, ST10) completing the panel of sequence types frequently involved in human bacteraemia [55]. We first verified that the wild-type strains strongly produced yersiniabactin, whereas both *irp2* mutants did not (Fig 2A). We then tested them in the mouse sepsis model and saw an increase in survival for both mutated strains (log-rank test p-value 0.02 and < 0.0001 or strain NILS46 and NILS9, respectively, Fig 2B and 2C, S4 Table) with no significant difference between the survival profiles for the two mutants (log-rank test p-value > 0.1). We therefore bring additional experimental evidence of the role of the HPI in extra-intestinal virulence. A much larger sample size would be required to evidence a dependency on genetic background for the relationship between HPI and virulence. Nevertheless, we have validated the causal link between the HPI and the virulence phenotype *in vivo* which demonstrates the power and accuracy of bacterial GWAS.

### High-throughput phenotypic data sheds light on HPI and other iron capture systems functions

**Fig 3:**
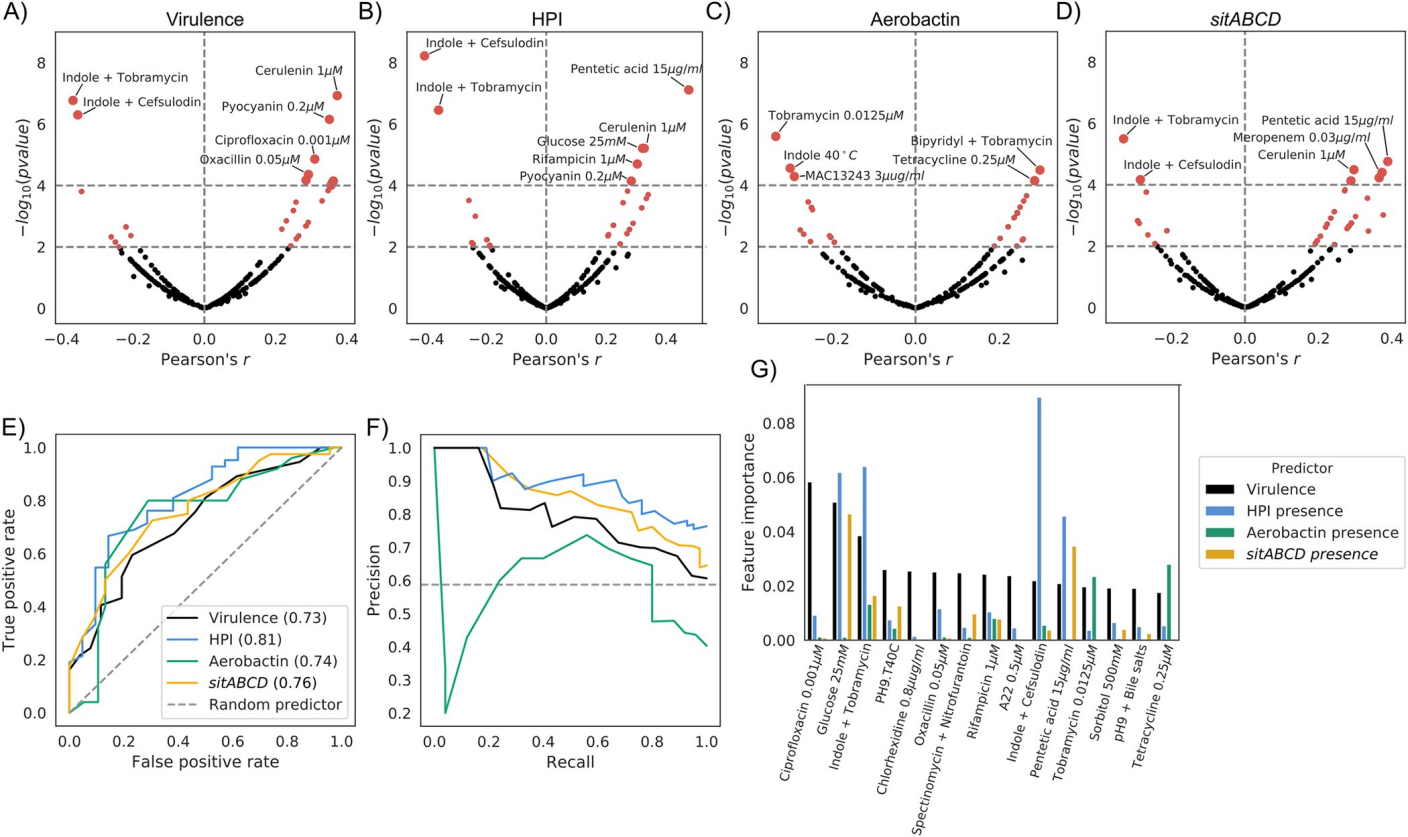
Growth profiles can predict virulence and presence of virulence factors. A-D) Volcano plots for the correlation between the strains’ growth profiles and: A) virulence levels, B) presence of the HPI, C) presence of aerobactin, and D) presence of *sitABCD*. E-F) Use of the strains’ growth profiles to build a predictor of virulence levels and presence of the three iron uptake systems. E) Receiver operating characteristic (ROC) curves and F) Precision-Recall curve for the four tested predictors. G) Feature importance for the predictors, showing the top 15 conditions contributing to the virulence level predictor.

The main function encoded by the HPI cassette is iron scavenging through the expression of the siderophore yersiniabactin [22], which has been previously validated in *E*. *coli* through knockout experiments [18]. The aerobactin operon also encodes an iron chelator [42], while the *sitABCD* operon encodes a Mn^2+^/Fe^2+^ ion transporter [43]. In order to investigate other putative functions of these operons and their relationship with virulence, we leveraged a previously-generated high-throughput phenotypic screening in an *E*. *coli* strain panel that largely overlaps with the strains used here (186 strains over 370 analyzed in this study) [28]. We observed a relatively strong correlation (Pearson’s correlation p-value < 1E-4) between growth profiles in certain in vitro conditions and both virulence and presence of the HPI, aerobactin and *sitABCD* operons (Fig 3A–3D, S5 Table).

As expected, we found a positive correlation between growth on the iron-sequestering agent pentetic acid [56] and both virulence and HPI/aerobactin/*sitABCD* presence (Pearson’s r: 0.36, 0.48, 0.23 and 0.39, respectively). We also found that growth in the presence of bipyridyl, an iron chelator, was positively correlated with the presence of aerobactin (exact condition: bipyridyl + tobramycin, Pearson’s r: 0.30). We similarly observed a positive correlation between growth with pyocyanin, a redox-active phenazine compound being able to reduce Fe^3+^ to Fe^2+^ [57], and both HPI/aerobactin/*sitABCD* presence (Pearson’s r: 0.35, 0.28, 0.26 and 0.27 respectively). All these mentioned growth conditions have a correlation sign that agrees with the iron scavenging function of the three gene clusters and their importance for virulence.

Interestingly, we also found similarly strong positive correlations between virulence and presence of iron capture systems with growth on sub-inhibitory concentrations of several antimicrobial agents, such as rifampicin, ciprofloxacin, tetracycline and ß-lactams such as amoxicillin, oxacillin, meropenem, cerulenin and colicin. These correlations might be due to the presence/absence of acquired resistance alleles and/or genes that are strongly associated with pathogenic strains, or might point to the role of iron homeostasis in intrinsic resistance to antibiotics [53]. To investigate these two hypotheses, we focused on tetracycline resistance, a common occurrence in the genus [34,55,58], and for which resistance genes can be easily found through sequence homology (Methods). We measured the correlation between the presence of tetracycline resistance genes, found in 26.8% of the strains, and virulence (Pearson’s r: 0.16), as well with the presence of either of the three iron capture systems (Pearson’s r: 0.21, 0.33 and 0.24 for HPI, aerobactin and *sitABCD*, respectively), which we found to be comparable in terms of sign and magnitude with the direct correlation between growth on sub-inhibitory concentration of tetracycline and the presence of resistance genes (Pearson’s r: 0.4). These correlations between virulence, iron capture systems and growth in the presence of tetracycline are however greatly reduced (Pearson’s r < 0.1) when correcting for the presence of tetracycline resistance genes using partial correlation. This suggests that there might not be a direct relationship between virulence, the GWAS hits and growth in the presence of tetracycline.

On the other hand we found that growth in presence of indole at 2 mM either in association with sub-inhibitory concentrations of cefsulodin and tobramycin, or alone at 40°C was negatively correlated with both virulence and HPI/aerobactin/*sitABCD* presence. Similar negative correlation was observed with aerobactin presence and the MAC13243 compound that increases outer membrane permeability [59]. This indicates that there might be a trade-off between growth in these conditions and virulence, *i*.*e*. virulent strains are less fit when growing in the presence of these compounds.

Given the relatively large number of conditions correlated with both virulence and presence of iron uptake systems, we tested whether these features could be predicted from growth profiles. We used the commonly-used random forests machine learning algorithm with appropriate partitioning of input data into training and test sets to tune hyperparameters and reduce overfitting (Methods). We trained and tested four classifiers for virulence and presence of the HPI, aerobactin and *sitABCD* operons with high predictive power, with the exception of aerobactin, which performed slightly worse, although still better than an empirical random (Fig 3E and 3F, S5 Fig and Methods). We noted that prediction of the gene clusters presence performs slightly better than virulence, possibly reflecting the complex nature of the latter phenotype. As expected, we found that conditions with relatively high correlation with each feature have a higher weight across classifiers (Fig 3G, S6 Table), which suggests that a subset of phenotypic tests might be sufficient to classify pathogenic strains. These results show how phenotypic data can be used to generate hypotheses for the function of virulence factors.

## Discussion

With the steady decline in the price of genomic sequencing and the increasing availability of molecular and phenotypic data for bacterial isolates, it has finally become possible to use statistical genomics approaches such as GWAS to uncover the genetic determinants of relevant phenotypes. Such approaches have the advantage of being unbiased, and can then be used to confirm previous targeted findings and potentially uncover new factors, given sufficient statistical power. The accumulation of other molecular and phenotypic data can on the other hand uncover variables correlated with phenotype, which can be used to generate testable hypotheses on the function of genomic hits and their role for growth in those correlated conditions. Given the rise of both *E*. *coli* extra-intestinal infections and antimicrobial resistance, we reasoned that the intrinsic virulence assessed in a calibrated mouse model of sepsis [14,27] is a phenotype worth exploring with such an unbiased approach.

Our work points to the fundamental role of iron scavenging in the extra-intestinal virulence phenotype in the genus *Escherichia* [60]. In fact, we found that 6 over the 7 GO terms significantly enriched were related to iron homeostasis. We were able to confirm earlier reports on the importance of the presence of the HPI in extra-intestinal virulence [18–20,22,54,61], which showed the strongest signal in both the unitigs and accessory genome association analysis, and whose importance was validated *in vitro* and in an *in vivo* model of virulence. The distribution of the HPI within the species resulting from multiple horizontal gene transfers via homologous recombination [62] has probably facilitated its identification using GWAS, since these methods favor the discovery of elements that are independently acquired across clades. We associated additional genetic factors to intrinsic virulence, such as the presence of the aerobactin and *sitABCD* operons, both related to iron scavenging together with the HPI. We also found mutations in core genes such as *hprRS* and *msrPQ* to be associated with virulence, whose role in response to oxidative stress and protein repair is compelling, although their association to virulence might be due to their physical proximity to the HPI. Thus, genetic variants in these genes could be associated with virulence through hitchhiking [62]. Hits in other core genes such as *rspB*, related to starvation sensing are similarly compelling. *rspB* is part of an operon with *rspA*, a gene encoding a protein involved in the degradation of homoserine lactone that signals starvation [63]. Further genetic and molecular characterization might elucidate the role of these core genes’ variants in extra-intestinal virulence. Additional factors might have been overlooked by this analysis, due to the relatively small sample size; we however estimate that those putative additional factors might have a relatively low penetrance, based on our simulations in an independent dataset. As sequencing of bacterial isolates is becoming more common in clinical settings [64–66], we expect to be able to uncover these additional genetic factors in future studies.

The association between both the intrinsic virulence phenotype and the presence of the virulence factors—such as the HPI—and previously collected growth data allowed us to generate hypotheses on mechanism of pathogenesis and putative additional functions of these factors. In particular we observed a strong correlation between growth on various antimicrobial agents and both virulence and HPI/aerobactin/*sitABCD* presence, which may be the result of the acquisition of both resistance genes/alleles and iron capture genes in these isolates, as exemplified for tetracycline resistance genes. This could be explained by a greater exposure to antibiotics and subsequent selection of resistance in clinical virulent strains, leading to the positive correlation we have observed. As such there might not be a causal relationship between increased iron uptake and antimicrobial resistance, but rather the two phenotypes coincide because of their selective advantage in the context of extra-intestinal pathogenesis.

The negative correlation between virulence and iron capture systems and growth profiles in the presence of 2 mM indole associated with stress conditions such as sub-lethal doses of specific antibiotics (cefsulodin and tobramycin) or high temperature but not indole alone, points however to the possible deleterious role of iron in such conditions. In *E*. *coli* cells grown in lysogeny broth in planktonic [67] or biofilm [68] conditions, sub-lethal concentrations of numerous antibiotics (ampicillin, trimethoprim, nalidixic acid, rifampicin, kanamycin and streptomycin) increase the endogenous production of indole to 1.5–6 mM. The production of indole is dependent on the amount of exogenous tryptophan, and it is conceivable that this range of indole concentrations obtained *in vitro* can be produced in the mammalian host [69] Indole is toxic for the cells above 3–5 mM, as it induces the production of reactive oxygen species and prevents cell division by modulating membrane potential [70,71]. A vicious circle is rapidly established as antibiotics increase the production of indole [67], which in turn destabilises the membrane [70,71], further increasing the penetration of the antibiotics. The toxicity of indole has been shown to be partly iron mediated due to the Fenton reaction, the deletion of TonB, an iron transporter, increasing resistance to the antibiotic [72]. Sub-lethal doses of tobramycin leads to an increase of reactive oxygen species in the bacterial cell in relation to intra-cellular iron and the Fenton reaction [73]. Thus, cells with increased import of extracellular iron might be more sensitive to sub-lethal doses of specific antibiotics, suggesting a potential “collateral sensitivity” related to both intrinsic virulence and the presence of the iron uptake systems. The expression “collateral sensitivity” is normally used to refer to selection for one antibiotic resistance resulting in increased sensitivity to a second antibiotic [74]. Here we propose to extend its meaning to include the negative correlation observed in this study; that is, the trade-off between the benefits brought by iron scavenging systems in one trait (virulence) being linked to detrimental changes in other traits (antibiotic sensitivity). Altogether, these data bring new light on the “liaisons dangereuses” between iron and antibiotics that could potentially be targeted [75]. More generally, they show that the presence of iron capturing systems can be either advantageous or disadvantageous, depending on the growth conditions. Further studies will however be needed to confirm this proposed “collateral sensitivity” and its molecular mechanism.

In conclusion, we showed the power of bacterial GWAS to identify major virulence determinants in bacteria. Within the *Escherichia* genus, iron capture systems seem to be the main predictors of the intrinsic extra-intestinal virulence, at least according to the mouse model of sepsis used here. Furthermore, this analysis demonstrates how a data-centric approach can increase our knowledge of complex bacterial phenotypes and guide future empirical work on gene function and its relationship to intrinsic virulence.

## Materials and methods

### Strains used

The full list of the 370 strains used in the association analysis, together with their main characteristics is reported in S1 Table. These strains belong to various published collections: ECOR (N = 71) [31], IAI (n = 81) [14], NILS (N = 82) [33], Septicoli (N = 39) [10], ROAR (N = 30) [34], Guyana (N = 12) [32], Coliville (N = 8) [35], FN (N = 6) [36], COLIRED (N = 3) [37], COLIBAFI (N = 2) [7], correspond to archetypal strains (N = 7) or are miscellaneous strains from our personal collections (N = 29). The isolation host is predominantly humans (N = 291), followed by animals (N = 72) and some strains were isolated from the environment (N = 6). One hundred and seventy strains were commensal whereas five and 187 were responsible of intestinal and extra-intestinal infections, respectively. The genomes of 295 strains were previously available, while the remaining 75 were sequenced as part of this work by Illumina technology as described previously [37]. The genome sequences of all strains are available through Figshare [76].

The construction of the *irp2* deletion mutants of the NILS9 and NILS46 strains was achieved following a strategy adapted from Datsenko and Wanner [77]. Primers used in the study are listed in S7 Table. In brief, primers used for gene disruption included 44–46 nucleotide homology extensions to the 5’- and 3’ regions of the target gene, respectively, and additional 20 nucleotides of priming sequence for amplification of the resistance cassette on the template plasmids pKD4. The PCR product was then transformed into strains carrying the helper plasmid pKOBEG expressing the lambda red recombinase under control of an arabinose-inducible promoter [78]. Kanamycin resistant transformants were selected and further screened for correct integration of the resistance marker by PCR. For elimination of the antibiotic resistance gene, helper plasmid pCP20 was used according to the published protocol. PCR followed by Sanger sequencing of the mutants were performed to verify the deletion and the presence of the expected scar.

### Yersiniabactin detection assay

Production of the siderophore yersiniabactin was detected and quantified using a luciferase reporter assay as described elsewhere [18,79]. Briefly, bacterial strains were cultivated in NBD medium for 24 hours at 37°C. Next, bacteria were pelleted by centrifugation and the supernatant was added to the indicator strain WR 1542 harbouring plasmid pACYC5.3L. All the genes necessary for yersiniabactin uptake are located on the plasmid pACYC5.3L, i.e. *irp6*, *irp7*, *irp8*, *fyuA*, *ybtA*. Furthermore, this plasmid is equipped with a fusion of the *fyuA* promoter region with the luciferase reporter gene. The amount of yersiniabactin can be quantified semi-quantitatively, as yersiniabactin-dependant upregulation of *fyuA* expression is determined by luciferase activity of the *fyuA-luc* reporter fusion.

### Mouse virulence assay

Ten female mice OF1 of 14–16 g (4 week-old) from Charles River (L'Arbresle, France) received a subcutaneous injection of 0.2 ml of bacterial suspension in the neck (2·10^8^ colony forming unit). Time to death was recorded during the following 7 days. Mice surviving more than 7 days were considered cured and sacrificed^14^. In each experiment, the *E*. *coli* CFT073 strain was used as a positive control killing all the inoculated mice whereas the *E*. *coli* K-12 MG1655 strain was used as a negative control for which all the inoculated mice survive [27]. The data were available for 134 strains from our previous works whereas the remaining 236 strains were tested in this study (S1 Table). For the mutant assays, 20 mice per strain were used to obtain statistical relevant data. The data was analysed using the lifeline package v0.21.0 [80].

### Association analysis

All genome-wide association analysis were carried out using pyseer, version v1.3.4 [25]. All input genomes were re-annotated using prokka, version v1.14.5 [81], to ensure uniform gene calls and excluding contigs whose size was below 200 base pairs. The core genome phylogenetic tree was generated using ParSNP [82] to generate the core genome alignment and gubbins v2.3.5 [83] to generate the phylogenetic tree. The strain’s pangenome was estimated using roary v3.13.0 [84]. Unitigs distributions from the input genome assemblies were computed using unitig-counter v1.0.5. The association between both unitigs and gene presence/absence patterns (“pangenome”) and phenotype (expressed as number of mice killed post-infection) was carried out using the FastLMM [85] linear mixed-model implemented in pyseer, using a kinship matrix derived from the phylogenetic tree as population structure. For both association analysis we used the number of unique presence/absence patterns to derive an appropriate multiple-testing corrected p-value threshold for the association likelihood ratio test (2.16E^-08^ and 5.45E^-06^ for the unitigs and pangenome analysis, respectively). Unitigs significantly associated with the phenotype were mapped back to each input genome using bwa mem v0.7.17-r1188 [86] and betools v.2.29.2 [87], using the pangenome analysis to collapse gene hits to individual groups of orthologs. A sample protein sequence for each groups of orthologs where at least one unitig with size 20 or higher was mapped was extracted giving priority to strain IAI39 when available, given it was the only strain with a complete genome available [88]; those sample sequences were used to search for homologs in the uniref50 database from uniprot [89] using blast v2.9.0 [90]. Each group of orthologs was then given a gene name using both available literature information and the results of the homology search. GO terms annotations were determined by submitting the protein sequence of each gene with associated unitigs to the eggnog-mapper website. GO terms enrichment was determined using goatools v1.0.6 [91]. Those genes with associated unitigs mapped to them and frequency in the pangenome > 0.9 were termed “core genes”; we searched for those genes in the E. coli K-12 genome (RefSeq: NC_000913.3) using blast v2.9.0 [90]

### Power simulations

Statistical power was estimated using a non-overlapping set of 548 complete *E*. *coli* genomes downloaded from NCBI RefSeq using ncbi-genome-download v0.2.9 on May 24th 2018. Each genome was subject to the same processing as the actual ones used in the real analysis (re-annotation, phylogenetic tree construction, pangenome estimation). The gene presence/absence patterns were used to run the simulations, in a similar way as described in the original SEER implementation [24]. Briefly, for each sample size, a random subset of strains was selected, and the likelihood ratio test p-value threshold was estimated by counting the number of unique gene presence/absence patterns in the reduced roary matrix. For each odds ratio tested, a binary case-control phenotype vector was simulated for the strains subset using the following formulae:

$$P_{case\vee variant}=\frac{D_{e}}{MAF}$$

$$P_{case\vee novariant}=\frac{\frac{S_{r}}{S_{r}+1}-D_{e}}{1-MAF}$$

Were *S_r_* is the ratio of case/controls (set at 1 in these simulations), *MAF* as the minimum allele frequency of the target gene in the strains subset, and *D_e_* the number of cases. pyseer’s LMM model was then applied to the actual presence/absence vector of the target gene and the likelihood ratio test p-value was compared with the empirical threshold, using the same population structure correction as the real analysis. The randomization was repeated 20 times for each gene and power was defined as the proportion of randomizations for each sample size and odds ratio whose p-value was below the threshold. To account for the influence of allele frequency on statistical power we picked 5 random genes for each allele frequency bin in the range [0.1–0.9].

### Correlations with growth profiles

The previously generated phenotypic data [28] for 186 strains over 370 total were used to compute correlations with both the number of mice killed after infection and presence/absence of the associated virulence factors. The data was downloaded from the ecoref website (https://evocellnet.github.io/ecoref/download/) and the pearson correlation with the s-scores (*i*.*e*. the normalized growth score for each strain in each condition [92]) was computed together with the correlation p-value. Prediction of tetracycline resistance was carried out using staramr v0.7.1 with the ResFinder database [93]. Four predictors, one for virulence (number of killed mice post-infection) and one for presence of the HPI, aerobactin and the *sitABCD* operon were built using the random forest classifier algorithm implemented in scikit-learn v.022.0 [94], using the s-scores as predictors. The input was column imputed, and 33% of the observations were kept as a test dataset, using a “stratified shuffle split” strategy. The remainder was used to train the classifier, using a grid search to select the number of trees and the maximum number of features used, through 10 rounds of stratified shuffle split with validation set size of 33% the training set and using the F1 measure as score. The performance of the classifiers on the test set were assessed by computing the area under the receiver operating characteristic curve (ROC-curve). For each predictor we derived the expected random baseline empirically by constructing a set of 15 predictors by shuffling the labels of the target vector, and keeping the training pipeline the same. We pooled the 15 random predictors and derived the average ROC and precision-recall curves with a 95% confidence interval.

### Software libraries

Code is mostly based on the Python programming language and the following libraries: numpy v1.17.3 [95], scipy v1.4.0 [96], biopython v1.75 [97,98], pandas v0.25.3 [99], pybedtools v0.8.0 [100], dendropy 4.4.0 [101], ete3 v3.1.1 [102], statsmodels v0.10.2 [103], matplotlib v3.1.2 [104], seaborn v0.9.0 [105], jupyterlab v1.2.4 [106] and snakemake v5.8.2 [107].

### Ethics statement

All animal experimentations were conducted following European (Directive 2010/63/EU on the protection of animals used for scientific purposes) and national recommendations (French Ministry of Agriculture and French Veterinary Services, accreditation A 75-18-05). The protocol was approved by the Animal Welfare Committee of the Veterinary Faculty in Lugo, University of Santiago de Compostela (AE-LU-002/12/INV MED.02/OUTROS 04).

## Supporting information

S1 Fig Simulations of statistical power on a non-overlapping set of complete *E*. *coli* genomes, using the 5 random genes for each frequency bin, repeating the simulation 20 times for each gene and odds ratio. The shaded area indicates the 95% confidence interval. The dotted red line indicates the sample size used in the actual analysis. AF, allele frequency.
(TIFF)

S2 Fig HPI structure conservation across strains. One strain per phylogroup or species is shown, using the same color scheme as Fig 1E for each gene.
(TIFF)

S3 Fig Location of core genome genes with associated unitigs mapped to them (red) with respect to the High Pathogenicity Island (HPI, black). The genome annotation of strain IAI39 is used as reference. Gene names were derived from *E*. *coli* K-12.
(TIFF)

S4 Fig Presence/absence patterns of known virulence factors. Solid color indicates presence, light grey indicates absence. Phenotypes (number of killed mice) and phylogroup or species of each strain are reported as in Fig 1A. “Other virulence factors” are (from inside the ring towards the outside): *sfaD*, *sfaE*, *ompT*, *traT*, *hra2*, *papC*, *iha*, *ireA*, *neuC*, *hlyC*, *clbQ* and *cnf1*.
(TIFF)

S5 Fig Empirical random predictors for virulence and the presence of iron capture systems from high-throughput growth data. Each line except the “Random predictor” represents the mean of 15 predictors built with suffled labels for the target variable. Vertical bars represent the 95% confidence interval.
(TIFF)

S1 Table Strains’ information, including virulence phenotype. (XLSX)

S2 Table Summary of the 81 genes with at least one mapped unitig. (XLSX)

S3 Table GO terms enrichment analysis for the 81 genes with at least one mapped unitig. (XLSX)

S4 Table Survival analysis for NILS9 and NILS46 wild-type and HPI mutants. (XLSX)

S5 Table Correlation between growth on stress conditions (s-scores) and both virulence and presence of the HPI. (XLSX)

S6 Table Feature importance for each growth condition in the random forests predictor for virulence and HPI presence. (XLSX)

S7 Table List of PCR primers used in this study. (XLSX)

## References

[1] Tenaillon O, Skurnik D, Picard B, Denamur E. The population genetics of commensal Escherichia coli. Nat. Rev. Microbiol. 2010;8:207–217. 10.1038/nrmicro2298 20157339

[2] Croxen MA, Brett Finlay B. Molecular mechanisms of Escherichia coli pathogenicity. Nature Reviews Microbiology. 2010;8:26–38. 10.1038/nrmicro2265 19966814

[3] Oaks JL, Besser TE, Walk ST, Gordon DM, Beckmen KB, Burek KA, et al Escherichia albertii in wild and domestic birds. Emerg. Infect. Dis. 2010;16:638–46. 10.3201/eid1604.090695 20350378 PMC3321939

[4] Clermont O, Gordon DM, Brisse S, Walk ST, Denamur E. Characterization of the cryptic Escherichia lineages: rapid identification and prevalence. Environ. Microbiol. 2011;13:2468–2477. 10.1111/j.1462-2920.2011.02519.x 21651689

[5] Blyton MDJ, Pi H, Vangchhia B, Abraham S, Trott DJ, Johnson JR, et al. Genetic Structure and Antimicrobial Resistance of Escherichia coli and Cryptic Clades in Birds with Diverse Human Associations. Appl. Environ. Microbiol. 2015;81:5123–5133. 10.1128/AEM.00861-15 26002899 PMC4495204

[6] Russo TA, Johnson JR. Medical and economic impact of extraintestinal infections due to Escherichia coli: focus on an increasingly important endemic problem. Microbes Infect. 2003;5:449–456. 10.1016/s1286-4579(03)00049-2 12738001

[7] Lefort A, Panhard X, Clermont O, Woerther P-L, Branger C, Mentré F, et al Host Factors and Portal of Entry Outweigh Bacterial Determinants to Predict the Severity of Escherichia coli Bacteremia. Journal of Clinical Microbiology. 2011;49:777–783. 10.1128/JCM.01902-10 21177892 PMC3067752

[8] Burdet C, Clermont O, Bonacorsi S, Laouénan C, Bingen E, Aujard Y, et al Escherichia coli bacteremia in children: age and portal of entry are the main predictors of severity. Pediatr. Infect. Dis. J. 2014;33:872–879. 10.1097/INF.0000000000000309 25222308

[9] Abernethy JK, Johnson AP, Guy R, Hinton N, Sheridan EA, Hope RJ.Thirty day all-cause mortality in patients with Escherichia coli bacteraemia in England. Clin. Microbiol. Infect. 2015;21:251e1–8. 10.1016/j.cmi.2015.01.001 25698659

[10] de Lastours V, Laouénan C, Royer G, Carbonnelle E, Lepeule R, Esposito-Farèse M, et al Mortality in Escherichia coli bloodstream infections: antibiotic resistance still does not make it. J. Antimicrob. Chemother. 2020;75:2334–2343. 10.1093/jac/dkaa161 32417924

[11] Vihta K-D, Stoesser N, Llewelyn MJ, Phuong Quan T, Davies T, Fawcett NJ, et al Trends over time in Escherichia coli bloodstream infections, urinary tract infections, and antibiotic susceptibilities in Oxfordshire, UK, 1998–2016: a study of electronic health records. The Lancet Infectious Diseases. 2018;18:1138–1149. 10.1016/S1473-3099(18)30353-0 30126643 PMC7612540

[12] Cassini A, Högberg LD, Plachouras D, Quattrocchi A, Hoxha A, Simonsen GS, et al Attributable deaths and disability-adjusted life-years caused by infections with antibiotic-resistant bacteria in the EU and the European Economic Area in 2015: a population-level modelling analysis. Lancet Infect. Dis. 2019;19:56–66. 10.1016/S1473-3099(18)30605-4 30409683 PMC6300481

[13] Baudron CR, Panhard X, Clermont O, Mentré F, Fantin B, Denamur E, et al Escherichia coli bacteraemia in adults: age-related differences in clinical and bacteriological characteristics, and outcome. Epidemiology & Infection. 2014;142:2672–2683. 24559489 10.1017/S0950268814000211 PMC9151316

[14] Picard B, Garcia JS, Gouriou S, Duriez P, Brahimi N, Bingen E, et al The link between phylogeny and virulence in Escherichia coli extraintestinal infection. Infect. Immun. 1999;67:546–553. 10.1128/IAI.67.2.546-553.1999 9916057 PMC96353

[15] Johnson JR, Kuskowski M. Clonal origin, virulence factors, and virulence. Infection and immunity. 2000;68:424–425. 10636718 10.1128/iai.68.1.424-425.2000 PMC97155

[16] Tourret J, Diard M, Garry L, Matic I, Denamur E. Effects of single and multiple pathogenicity island deletions on uropathogenic Escherichia coli strain 536 intrinsic extra-intestinal virulence. Int. J. Med. Microbiol. 2010;300:435–439. 10.1016/j.ijmm.2010.04.013 20510652

[17] Ingle DJ, Clermont O, Skurnik D, Denamur E, Walk ST, Gordon DM, et al Biofilm formation by and thermal niche and virulence characteristics of Escherichia spp. Appl. Environ. Microbiol. 2011;77:2695–2700. 10.1128/AEM.02401-10 21335385 PMC3126365

[18] Smati M, Magistro G, Adiba S, Wieser A, Picard B, Schubert S, et al Strain-specific impact of the high-pathogenicity island on virulence in extra-intestinal pathogenic Escherichia coli. Int. J. Med. Microbiol. 2017;307:44–56. 10.1016/j.ijmm.2016.11.004 27923724

[19] Johnson JR, Russo TA. Molecular Epidemiology of Extraintestinal Pathogenic Escherichia coli. EcoSal Plus. 2018:8. 10.1128/ecosalplus.esp-0004-2017 PMC11575673 29667573

[20] Schubert S, Cuenca S, Fischer D, Heesemann J. High-pathogenicity island of Yersinia pestis in enterobacteriaceae isolated from blood cultures and urine samples: prevalence and functional expression. J. Infect. Dis. 2000;182:1268–1271. 10979932 10.1086/315831

[21] Paauw A, Leverstein-van Hall MA, van Kessel KPM., Verhoef J, Fluit AC. Yersiniabactin reduces the respiratory oxidative stress response of innate immune cells. PLoS One. 2009;4:e8240 10.1371/journal.pone.0008240 20041108 PMC2795162

[22] Schubert S, Picard B, Gouriou S, Heesemann J, Denamur E. Yersinia high-pathogenicity island contributes to virulence in Escherichia coli causing extraintestinal infections. Infect. Immun. 2002;70:5335–5337. 10.1128/iai.70.9.5335-5337.2002 12183596 PMC128248

[23] Earle SG, Wu C-H, Charlesworth J, Stoesser N, Gordon NC, Walker TM, et al Identifying lineage effects when controlling for population structure improves power in bacterial association studies. Nature Microbiology. 2016;1;1–8. 10.1038/nmicrobiol.2016.41 PMC5049680 27572646

[24] Lees JA, Vehkala M, Välimäki N, Harris SR, Chewapreecha C, Croucher NJ, et al Sequence element enrichment analysis to determine the genetic basis of bacterial phenotypes. Nat. Commun. 2016;7:12797 10.1038/ncomms12797 27633831 PMC5028413

[25] Lees J, Galardini M, Bentley SD, Weiser JN. pyseer: a comprehensive tool for microbial pangenome-wide association studies. bioRxiv. 2018. 10.1093/bioinformatics/bty539 PMC6289128 30535304

[26] Jaillard M, Lima L, Tournoud M, Mahé P, van Belkum A, Lacroix V, Jacob L, et al A fast and agnostic method for bacterial genome-wide association studies: Bridging the gap between k-mers and genetic events. PLoS Genet. 2018;14:e1007758 10.1371/journal.pgen.1007758 30419019 PMC6258240

[27] Johnson JR, Clermont O, Menard M, Kuskowski MA, Picard B, Denamur E, et al Experimental mouse lethality of Escherichia coli isolates, in relation to accessory traits, phylogenetic group, and ecological source. J. Infect. Dis. 2006;194:1141–1150. 10.1086/507305 16991090

[28] Galardini M, Koumoutsi A, Herrera-Dominguez L, Cordero Varela JA, Telzerow A, Wagih O, et al Phenotype inference in an Escherichia coli strain panel. Elife. 2017;6:1–19. 10.7554/eLife.31035 PMC5745082 29280730

[29] Power RA, Parkhill J, de Oliveira, T. Microbial genome-wide association studies: lessons from human GWAS. Nat. Rev. Genet. 2016;18:41–50. 10.1038/nrg.2016.132 27840430

[30] Clermont O, Christenson JK, Denamur E, Gordon DM. The Clermont Escherichia coli phylo-typing method revisited: improvement of specificity and detection of new phylo-groups. Environ. Microbiol. Rep. 2013;5:58–65. 10.1111/1758-2229.12019 23757131

[31] Ochman H, Selander RK. Standard reference strains of Escherichia coli from natural populations. J. Bacteriol. 1984;157:690–693. 10.1128/JB.157.2.690-693.1984 6363394 PMC215307

[32] Lescat M, Clermont O, Woerther PL, Glodt J, Dion S, Skurnik D, et al. Commensal Escherichia coli strains in Guiana reveal a high genetic diversity with host-dependant population structure. Environ. Microbiol. Rep. 2013;5:49–57. 10.1111/j.1758-2229.2012.00374.x 23757130

[33] Bleibtreu A, Clermont O, Darlu P, Glodt J, Branger C, Picard B, et al The rpoS gene is predominantly inactivated during laboratory storage and undergoes source-sink evolution in Escherichia coli species. J. Bacteriol. 2014;196:4276–4284. 10.1128/JB.01972-14 25266386 PMC4248845

[34] Skurnik D, Clermont O, Guillard T, Launay A, Danilchanka O, Pons S, et al Emergence of Antimicrobial-Resistant Escherichia coli of Animal Origin Spreading in Humans. Mol. Biol. Evol. 2016;33:898–914. 10.1093/molbev/msv280 26613786 PMC5013867

[35] Massot M, Daubié A-S, Clermont O, Jauréguy F, Couffignal C, Dahbi G, et al Phylogenetic, virulence and antibiotic resistance characteristics of commensal strain populations of Escherichia coli from community subjects in the Paris area in 2010 and evolution over 30 years. Microbiology. 2016;162:642–650. 10.1099/mic.0.000242 26822436 PMC6365622

[36] Nowrouzian FL, Clermont O, Edin M, Östblom A, Denamur E, Wold AE, et al Escherichia coli B2 Phylogenetic Subgroups in the Infant Gut Microbiota: Predominance of Uropathogenic Lineages in Swedish Infants and Enteropathogenic Lineages in Pakistani Infants. Appl. Environ. Microbiol. 2019;85. 10.1128/AEM.01681-19 PMC6881811 31562173

[37] Bourrel AS, Poirel L, Royer G, Darty M, Vuillemin X, Kieffer N, et al Colistin resistance in Parisian inpatient faecal Escherichia coli as the result of two distinct evolutionary pathways. J. Antimicrob. Chemother. 2019;74:1521–1530. 10.1093/jac/dkz090 30863849

[38] Moissenet D, Salauze B, Clermont O, Bingen E, Arlet G, Denamur E, et al Meningitis caused by Escherichia coli producing TEM-52 extended-spectrum beta-lactamase within an extensive outbreak in a neonatal ward: epidemiological investigation and characterization of the strain. J. Clin. Microbiol. 2010;48:2459–2463. 10.1128/JCM.00529-10 20519482 PMC2897521

[39] Clermont O, Dixit OVA, Vangchhia B, Condamine B, Dion S, Bridier‐Nahmias A, et al Characterization and rapid identification of phylogroup G in Escherichia coli, a lineage with high virulence and antibiotic resistance potential. Environ. Microbiol. 2019;21:3107–3117. 10.1111/1462-2920.14713 31188527

[40] Hacker J, Carniel E. Ecological fitness, genomic islands and bacterial pathogenicity. A Darwinian view of the evolution of microbes. EMBO Rep. 2001;2:376–381. 10.1093/embo-reports/kve097 11375927 PMC1083891

[41] Touchon M, Perrin A, Moura de Sousa JA, Vangchhia B, Burn S, O’Brien CL, et al Phylogenetic background and habitat drive the genetic diversification of *Escherichia coli*. PLoS Genet. 2020;16:e1008866 10.1371/journal.pgen.1008866 32530914 PMC7314097

[42] Warner PJ, Williams PH, Bindereif A, Neilands JB. ColV plasmid-specific aerobactin synthesis by invasive strains of Escherichia coli. Infect. Immun. 1981;33:540–545. 10.1128/IAI.33.2.540-545.1981 6456229 PMC350734

[43] Bearden SW, Staggs TM, Perry RD. An ABC transporter system of Yersinia pestis allows utilization of chelated iron by Escherichia coli SAB11. J. Bacteriol. 1998;180:1135–1147. 10.1128/JB.180.5.1135-1147.1998 9495751 PMC107000

[44] Mühldorfer I, Hacker J. Genetic aspects of Escherichia coli virulence. Microb. Pathog. 1994;16:171–181. 10.1006/mpat.1994.1018 7522300

[45] Graham AI, Hunt S, Stokes SL, Bramall N, Bunch J, Cox AG, et al Severe zinc depletion of Escherichia coli: roles for high affinity zinc binding by ZinT, zinc transport and zinc-independent proteins. J. Biol. Chem. 2009;284:18377–18389. 10.1074/jbc.M109.001503 19377097 PMC2709383

[46] Becker A-K, Zeppenfeld T, Staab A, Seitz S, Boos W, Morita T, et al YeeI, a novel protein involved in modulation of the activity of the glucose-phosphotransferase system in Escherichia coli K-12. J. Bacteriol. 2006;188:5439–5449. 10.1128/JB.00219-06 16855233 PMC1540043

[47] Whipp MJ, Camakaris H, Pittard AJ. Cloning and analysis of the shiA gene, which encodes the shikimate transport system of escherichia coli K-12. Gene.1998;209:185–192. 10.1016/s0378-1119(98)00043-2 9524262

[48] Prévost K, Salvail H, Desnoyers G, Jacques J-F, Phaneuf E, Massé E, et al The small RNA RyhB activates the translation of shiA mRNA encoding a permease of shikimate, a compound involved in siderophore synthesis. Mol. Microbiol. 2007;64:1260–1273. 10.1111/j.1365-2958.2007.05733.x 17542919

[49] Urano H, Yoshida M, Ogawa A, Yamamoto K, Ishihama A, Ogasawara H, et al Cross-regulation between two common ancestral response regulators, HprR and CusR, in Escherichia coli. Microbiology.2017;163:243–252. 10.1099/mic.0.000410 27983483

[50] Gennaris A, Ezraty B, Henry C, Agrebi R, Vergnes A, Oheix E, et al Repairing oxidized proteins in the bacterial envelope using respiratory chain electrons. Nature. 2015;528;409–412. 10.1038/nature15764 26641313 PMC4700593

[51] Ilbert M, Méjean V, Giudici-Orticoni M-T, Samama J-P, Iobbi-Nivol C. Involvement of a mate chaperone (TorD) in the maturation pathway of molybdoenzyme TorA. J. Biol. Chem. 2003;278:28787–28792. 10.1074/jbc.M302730200 12766163

[52] Méjean V, Lobbi‐Nivol C, Lepelletier M, Giordano G, Chippaux M, Pascal M-C. TMAO anaerobic respiration in Escherichia coli: involvement of the tor operon. Mol. Microbiol. 1994;11:1169–1179. 10.1111/j.1365-2958.1994.tb00393.x 8022286

[53] Diard M, Garry L, Selva M, Mosser T, Denamur R, Matic I, et al Pathogenicity-associated islands in extraintestinal pathogenic Escherichia coli are fitness elements involved in intestinal colonization. J. Bacteriol. 2010;192:4885–4893. 10.1128/JB.00804-10 20656906 PMC2944530

[54] Johnson JR, Magistro G, Clabots C, Porter S, Manges A, Thuras P, et al Contribution of yersiniabactin to the virulence of an Escherichia coli sequence type 69 (‘clonal group A’) cystitis isolate in murine models of urinary tract infection and sepsis. Microb. Pathog. 2018;120:128–131. 10.1016/j.micpath.2018.04.048 29702209

[55] Kallonen T, Brodrick HJ, Harris SR, Corander J, Brown NM, Martin V, et al. Systematic longitudinal survey of invasive Escherichia coli in England demonstrates a stable population structure only transiently disturbed by the emergence of ST131. Genome Res. (2017) 10.1101/gr.216606.116 28720578 PMC5538559

[56] Pippard MJ, Jackson MJ, Hoffman K, Petrou M, Modell, C. B. Iron chelation using subcutaneous infusions of diethylene triamine penta-acetic acid (DTPA). Scand. J. Haematol. 1986;36:466–472. 10.1111/j.1600-0609.1986.tb02282.x 3738427

[57] Cornelis P, Dingemans J. Pseudomonas aeruginosa adapts its iron uptake strategies in function of the type of infections. Front. Cell. Infect. Microbiol. 2013;3:75 10.3389/fcimb.2013.00075 24294593 PMC3827675

[58] Mazel D, Dychinco B, Webb VA, Davies J. Antibiotic resistance in the ECOR collection: integrons and identification of a novel aad gene. Antimicrob. Agents Chemother. 2000;44:1568–1574. 10.1128/aac.44.6.1568-1574.2000 10817710 PMC89914

[59] Muheim C, Götzke H, Eriksson AU, Lindberg S, Lauritsen I, Nørholm MHH, et al Increasing the permeability of Escherichia coli using MAC13243. Scientific Reports. 2017;7 http://paperpile.com/b/XWFpcJ/eEaYFhttp://paperpile.com/b/XWFpcJ/eEaYFhttp://paperpile.com/b/XWFpcJ/eEaYFhttp://paperpile.com/b/XWFpcJ/eEaYFhttp://paperpile.com/b/XWFpcJ/eEaYF 10.1038/s41598-017-00035-9 29247166 PMC5732295

[60] Skaar EP. The battle for iron between bacterial pathogens and their vertebrate hosts. PLoS Pathog. 2010;6:e1000949 10.1371/journal.ppat.1000949 20711357 PMC2920840

[61] Johnson JR. Johnston BD, Porter S, Thuras P, Aziz M, Price LB. Accessory Traits and Phylogenetic Background Predict Escherichia coli Extraintestinal Virulence Better Than Does Ecological Source. J. Infect. Dis. 2019;219:121–132. 10.1093/infdis/jiy459 30085181 PMC6284546

[62] Schubert S, Darlu P, Clermont O, Wieser A, Magistro G, Hoffmann C, et al Role of Intraspecies Recombination in the Spread of Pathogenicity Islands within the Escherichia coli Species. PLoS Pathog. 2009;5:e1000257 10.1371/journal.ppat.1000257 19132082 PMC2606025

[63] Huisman GW, Kolter R. Sensing starvation: a homoserine lactone—dependent signaling pathway in Escherichia coli. Science. 1994;265:537–539. 10.1126/science.7545940 7545940

[64] Fricke WF, Rasko DA. Bacterial genome sequencing in the clinic: bioinformatic challenges and solutions. Nat. Rev. Genet. 2014;15:49–55. 10.1038/nrg3624 24281148

[65] Quainoo S, Coolen JPM, van Hijum SAFT, Huynen MA, Melchers WJG, van Schaik W, et al. Whole-Genome Sequencing of Bacterial Pathogens: The Future of Nosocomial Outbreak Analysis. Clin. Microbiol. Rev. 2017;30:1015–1063. 10.1128/CMR.00016-17 28855266 PMC5608882

[66] Tagini F, Greub G. Bacterial genome sequencing in clinical microbiology: a pathogen-oriented review. Eur. J. Clin. Microbiol. Infect. Dis. 2017;36:2007–2020. 10.1007/s10096-017-3024-6 28639162 PMC5653721

[67] Mathieu A, Fleurier S, Frénoy A, Dairou J, Bredeche M-F, Sanchez-Vizuete P, et al Discovery and Function of a General Core Hormetic Stress Response in E. coli Induced by Sublethal Concentrations of Antibiotics. Cell Rep. 2016;17:46–57. 10.1016/j.celrep.2016.09.001 27681420

[68] Kuczyńska-Wiśnik D, Matuszewska E, Furmanek-Blaszk B, Leszczyńska D, Grudowska A, Szczepaniak P, et al Antibiotics promoting oxidative stress inhibit formation of Escherichia coli biofilm via indole signalling. Res. Microbiol. 2010;161:847–853. 10.1016/j.resmic.2010.09.012 20868745

[69] Li G, Young KD. Indole production by the tryptophanase TnaA in Escherichia coli is determined by the amount of exogenous tryptophan. Microbiology. 2013;159:402–410. 10.1099/mic.0.064139-0 23397453

[70] Garbe TR, Kobayashi M, Yukawa H. Indole-inducible proteins in bacteria suggest membrane and oxidant toxicity. Arch. Microbiol. 2000;173:78–82. 10.1007/s002030050012 10648109

[71] Chimerel C, Field CM, Piñero-Fernandez S, Keyser UF, Summers DK. Indole prevents Escherichia coli cell division by modulating membrane potential. Biochim. Biophys. Acta. 2012;1818:1590–1594. 10.1016/j.bbamem.2012.02.022 22387460 PMC3793866

[72] Giroux X, Su W-L, Bredeche M-F, Matic I. Maladaptive DNA repair is the ultimate contributor to the death of trimethoprim-treated cells under aerobic and anaerobic conditions. Proc. Natl. Acad. Sci. U. S. A. 2017;114:11512–11517. 10.1073/pnas.1706236114 29073080 PMC5664507

[73] Baharoglu Z, Krin E, Mazel D. RpoS plays a central role in the SOS induction by sub-lethal aminoglycoside concentrations in Vibrio cholerae. PLoS Genet. 2013;9:e1003421 10.1371/journal.pgen.1003421 23613664 PMC3623755

[74] Pál C, Papp B, Lázár V. Collateral sensitivity of antibiotic-resistant microbes. Trends Microbiol. 2015;23:401–407. 10.1016/j.tim.2015.02.009 25818802 PMC5958998

[75] Ezraty B, Barras F. The ‘liaisons dangereuses’ between iron and antibiotics. FEMS Microbiol. Rev. 2016;40:418–435. 10.1093/femsre/fuw004 26945776

[76] Galardini M. Escherichia coli pathogenicity GWAS: input genome sequences (updated). (2020) 10.6084/m9.figshare.11879340.v1

[77] Datsenko KA, Wanner BL. One-step inactivation of chromosomal genes in Escherichia coli K-12 using PCR products. Proc. Natl. Acad. Sci. U. S. A. 2000;97:6640–6645. 10.1073/pnas.120163297 10829079 PMC18686

[78] Chaveroche MK, Ghigo JM, d’Enfert C. A rapid method for efficient gene replacement in the filamentous fungus Aspergillus nidulans. Nucleic Acids Res. 2000;28:E97 10.1093/nar/28.22.e97 11071951 PMC113889

[79] Martin P, Marcq I, Magistro G, Penary M, Garcie C, Payros D, et al Interplay between Siderophores and Colibactin Genotoxin Biosynthetic Pathways in Escherichia coli. PLoS Pathogens. 2013;9:e1003437 10.1371/journal.ppat.1003437 23853582 PMC3708854

[80] Davidson-Pilon C, Kalderstam J, Zivich P, Kuhn B, Fiore-Gartland A, Moneda L, et al CamDavidsonPilon/lifelines: v0.21.0. 2019 10.5281/zenodo.2638135

[81] Seemann T. Prokka: rapid prokaryotic genome annotation. Bioinformatics. 2014;30:2068–2069. 10.1093/bioinformatics/btu153 24642063

[82] Treangen TJ, Ondov BD, Koren S, Phillippy AM. The Harvest suite for rapid core-genome alignment and visualization of thousands of intraspecific microbial genomes. Genome Biol. 2014;15:524 10.1186/s13059-014-0524-x 25410596 PMC4262987

[83] Croucher NJ, Page AJ, Connor TR, Delaney AJ, Keane JA, et al Rapid phylogenetic analysis of large samples of recombinant bacterial whole genome sequences using Gubbins. Nucleic Acids Res. 2015;43:e15 10.1093/nar/gku1196 25414349 PMC4330336

[84] Page AJ, Cummins CA, Hunt M, Wong VK, Reuter S, Holden MTG, et al Roary: rapid large-scale prokaryote pan genome analysis. Bioinformatics. 2015;31:3691–3693. 10.1093/bioinformatics/btv421 26198102 PMC4817141

[85] Lippert C, Listgarten J, Liu Y, Kadie CM, Davidson RI, Heckerman D, et al FaST linear mixed models for genome-wide association studies. Nature Methods. 2011;8:833–835. 10.1038/nmeth.1681 21892150

[86] Li H. Aligning sequence reads, clone sequences and assembly contigs with BWA-MEM. 2013. arXiv [q-bio.GN].

[87] Quinlan AR, Hall IM. BEDTools: a flexible suite of utilities for comparing genomic features. Bioinformatics. 2010;26:841–842. 10.1093/bioinformatics/btq033 20110278 PMC2832824

[88] Touchon M, Hoede C, Tenaillon O, Barbe V, Baeriswyl S, Bidet P, et al Organised genome dynamics in the Escherichia coli species results in highly diverse adaptive paths. PLoS Genet. 2009;5:e1000344 10.1371/journal.pgen.1000344 19165319 PMC2617782

[89] Consortium UniProt. UniProt: a hub for protein information. Nucleic Acids Res. 2015;43:D204–12. 10.1093/nar/gku989 25348405 PMC4384041

[90] Altschul SF, Gish W, Miller W, Myers EW, Lipman DJ. Basic local alignment search tool. Journal of Molecular Biology. 1990;215:403–410. 10.1016/S0022-2836(05)80360-2 2231712

[91] Klopfenstein DV, Zhang L, Pedersen BS, Ramírez F, Warwick Vesztrocy A, Naldi A, et al. GOATOOLS: A Python library for Gene Ontology analyses. Sci. Rep. 2018;8:10872 10.1038/s41598-018-28948-z 30022098 PMC6052049

[92] Collins SR, Schuldiner M, Krogan NJ, Weissman JS. A strategy for extracting and analyzing large-scale quantitative epistatic interaction data. Genome Biol. 2006;7:R63 10.1186/gb-2006-7-7-r63 16859555 PMC1779568

[93] Zankari E, Hasman H, Cosentino S, Vestergaard M, Rasmussen S, Lund O, et al Identification of acquired antimicrobial resistance genes. J. Antimicrob. Chemother. 2012;67:2640–2644. 10.1093/jac/dks261 22782487 PMC3468078

[94] Pedregosa F, Varoquaux G, Gramfort A, Michel V, Thirion B, Grisel O, et al Scikit-learn: Machine Learning in Python. J. Mach. Learn. Res. 2011;12:2825–2830.

[95] Van Der Walt S, Colbert SC, Varoquaux G. The NumPy array: a structure for efficient numerical computation. Comput. Sci. Eng. 2011;13:22–30.

[96] Jones E, Oliphant T, Peterson P. SciPy: Open source scientific tools for Python. 2001 http://www.scipy.org/.

[97] Cock PJA, Antao T, Chang JT, Chapman BA, Cox CJ, Dalke A, et al Biopython: freely available Python tools for computational molecular biology and bioinformatics. Bioinformatics. 2009;25:1422–1423. 10.1093/bioinformatics/btp163 19304878 PMC2682512

[98] Talevich E, Invergo BM, Cock PJ, Chapman B. a. Bio.Phylo: A unified toolkit for processing, analyzing and visualizing phylogenetic trees in Biopython. BMC Bioinformatics. 2012;13:209 10.1186/1471-2105-13-209 22909249 PMC3468381

[99] McKinney W, Others. Data structures for statistical computing in Python. in *Proceedings of the 9th Python in Science Conference* vol. 2010;445:51–56.

[100] Dale RK, Pedersen BS, Quinlan AR. Pybedtools: a flexible Python library for manipulating genomic datasets and annotations. Bioinformatics. 2011;27:3423–3424. 10.1093/bioinformatics/btr539 21949271 PMC3232365

[101] Sukumaran J, Holder MT. DendroPy: a Python library for phylogenetic computing. Bioinformatics. 2010;26:1569–1571. 10.1093/bioinformatics/btq228 20421198

[102] Huerta-Cepas J, Serra F, Bork P. ETE 3: Reconstruction, Analysis, and Visualization of Phylogenomic Data. Mol. Biol. Evol. 2016;33:1635–1638. 10.1093/molbev/msw046 26921390 PMC4868116

[103] Seabold S, Perktold J. Statsmodels: Econometric and statistical modeling with python. in Proceedings of the 9th Python in Science Conference vol. 57 61; SciPy society Austin, 2010.

[104] Hunter JD. Matplotlib: A 2D Graphics Environment. Computing in Science Engineering. 2007;9:90–95.

[105] Waskom M, Botvinnik O, O'Kane D, Hobson P, Ostblom J, Lukauskas S, et al. mwaskom/seaborn: v0.9.0 (July 2018). 2018. 10.5281/zenodo.1313201

[106] Kluyver T, Ragan-Kelley B, Pérez F, Granger B, Bussonnier M, Frederic J, et al Jupyter Notebooks-a publishing format for reproducible computational workflows. in ELPUB 87–90. 2016.

[107] Köster J, Rahmann S. Snakemake-a scalable bioinformatics workflow engine. Bioinformatics. 2018;34:3600 10.1093/bioinformatics/bty350 29788404

