# Supplementary figures and images for: Major role of iron uptake systems in the intrinsic extra-intestinal virulence of the genus Escherichia revealed by a genome-wide association study
Source: PLoS Genet. 2020 Oct 28;16(10):e1009065. doi: 10.1371/journal.pgen.1009065 (PMC7592755; doi:10.1371/journal.pgen.1009065)

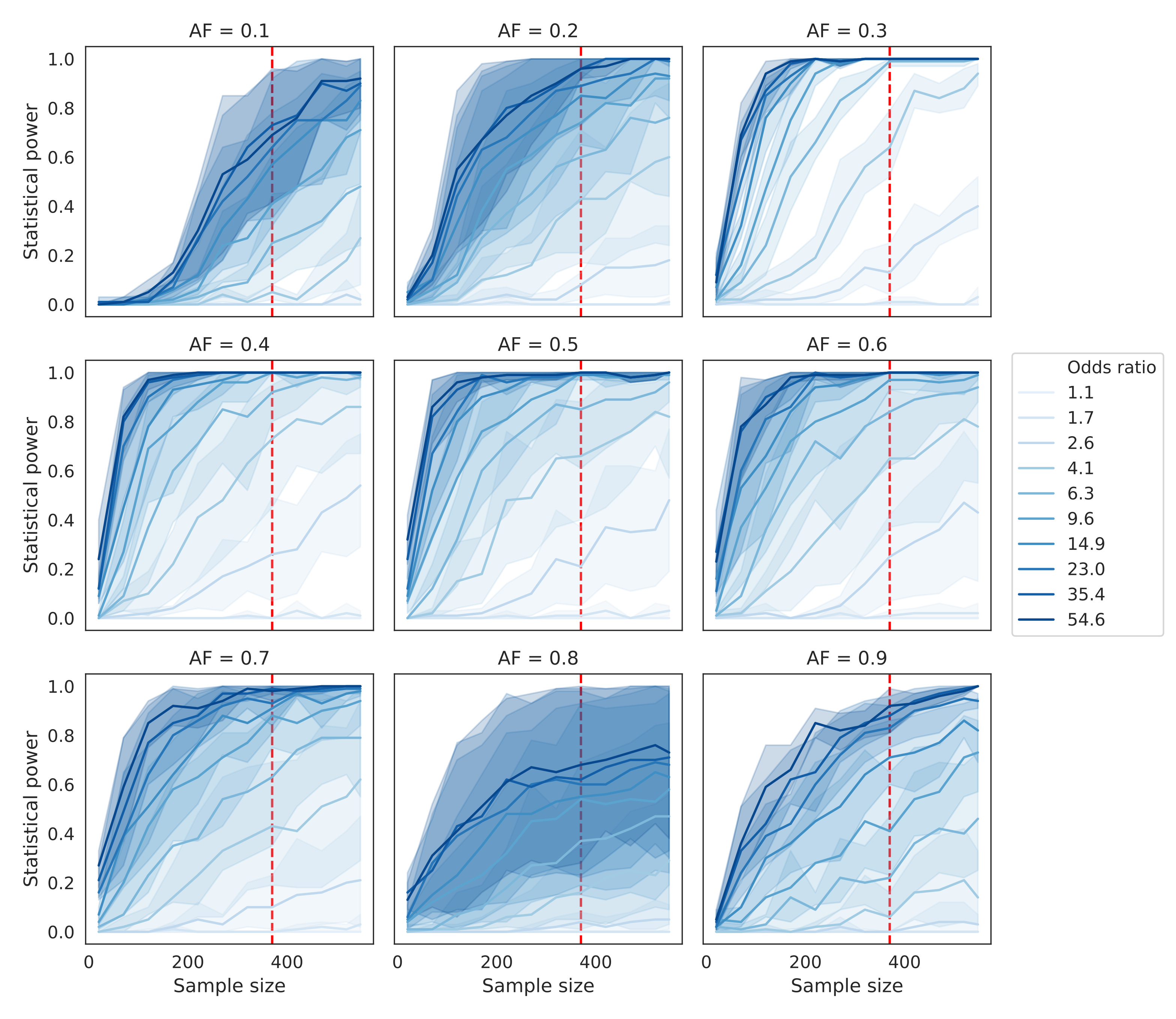

Supplement: S1 Fig — The shaded area indicates the 95% confidence interval. The dotted red line indicates the sample size used in the actual analysis. AF, allele frequency. (TIFF) [file pgen.1009065.s001.tiff]

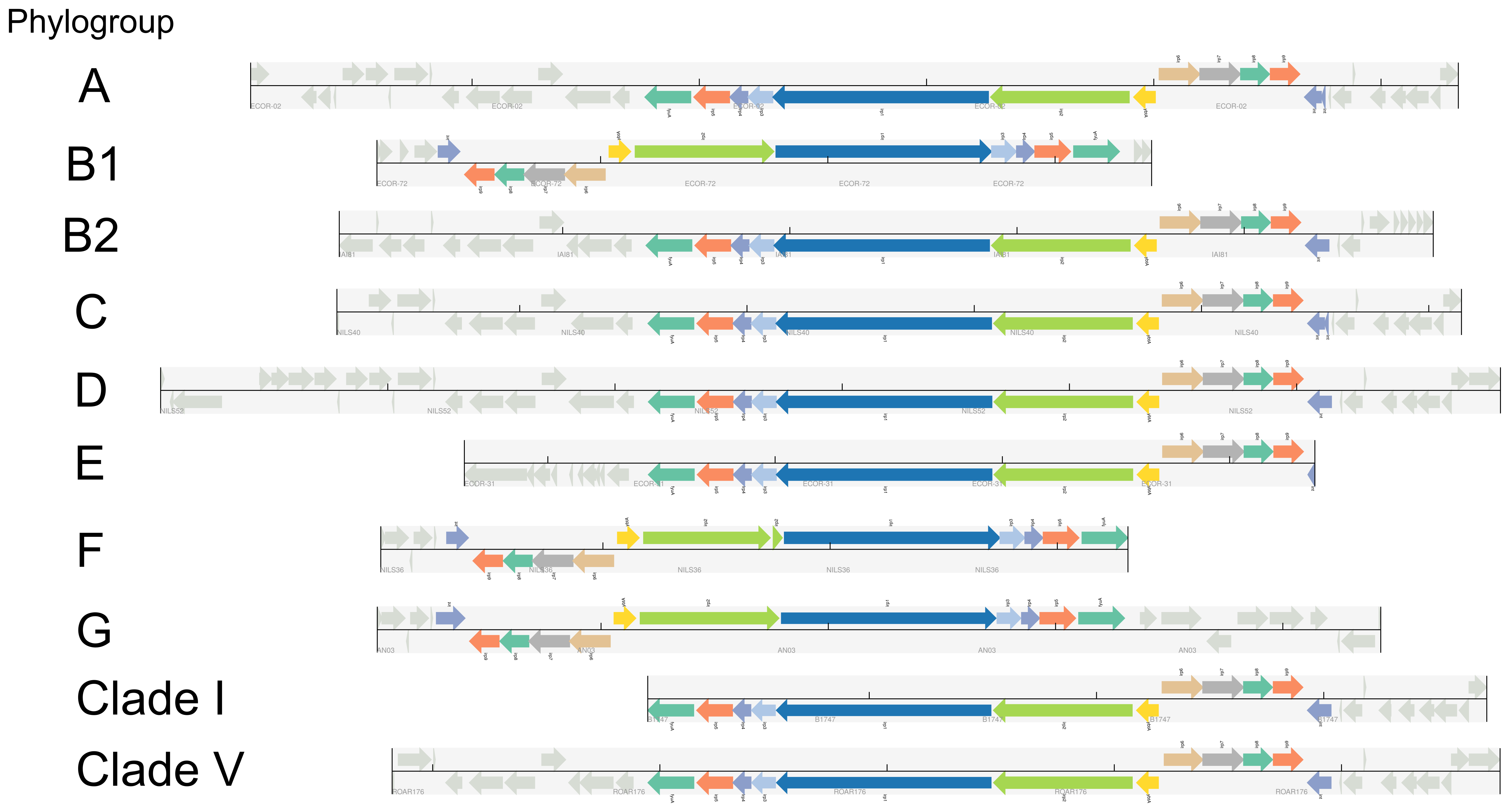

Supplement: S2 Fig — One strain per phylogroup or species is shown, using the same color scheme as Fig 1E for each gene. (TIFF) [file pgen.1009065.s002.tiff]

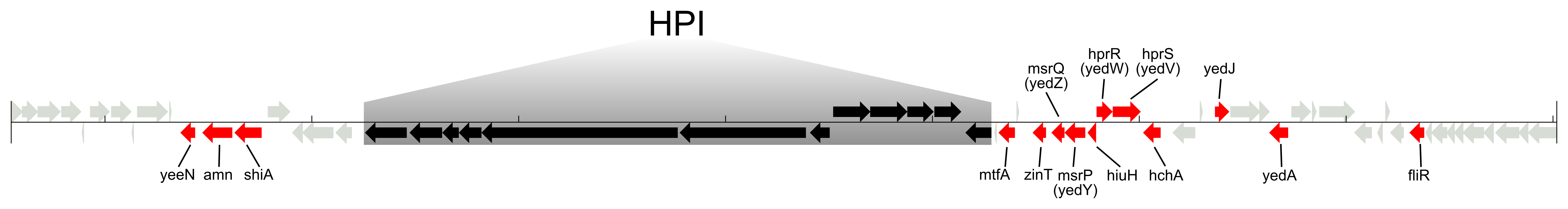

Supplement: S3 Fig — The genome annotation of strain IAI39 is used as reference. Gene names were derived from E. coli K-12. (TIFF) [file pgen.1009065.s003.tiff]

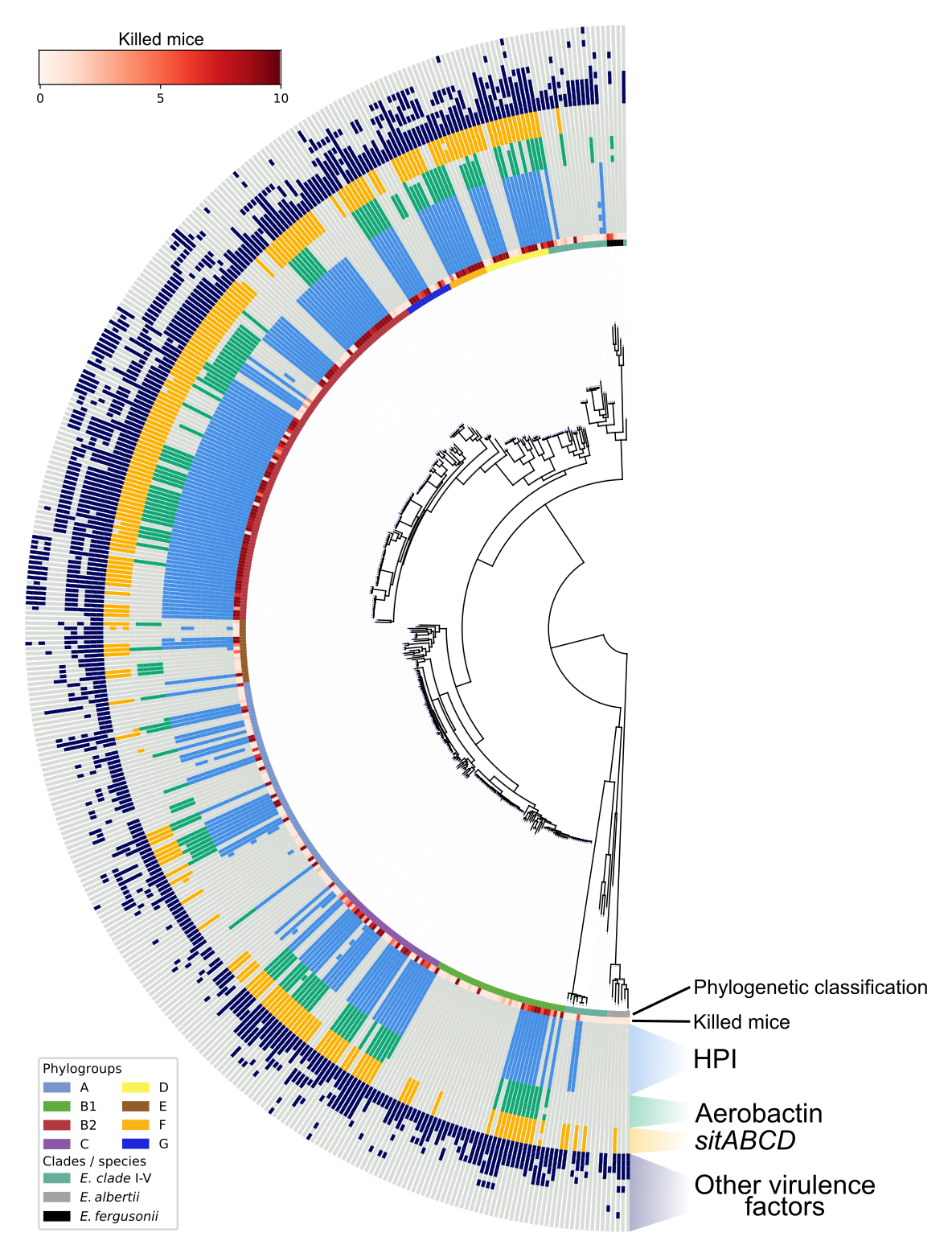

Supplement: S4 Fig — Solid color indicates presence, light grey indicates absence. Phenotypes (number of killed mice) and phylogroup or species of each strain are reported as in Fig 1A. “Other virulence factors” are (from inside the ring towards the outside): sfaD, sfaE, ompT, traT, hra2, papC, iha, ireA, neuC, hlyC, clbQ and cnf1. (TIFF) [file pgen.1009065.s004.tiff]

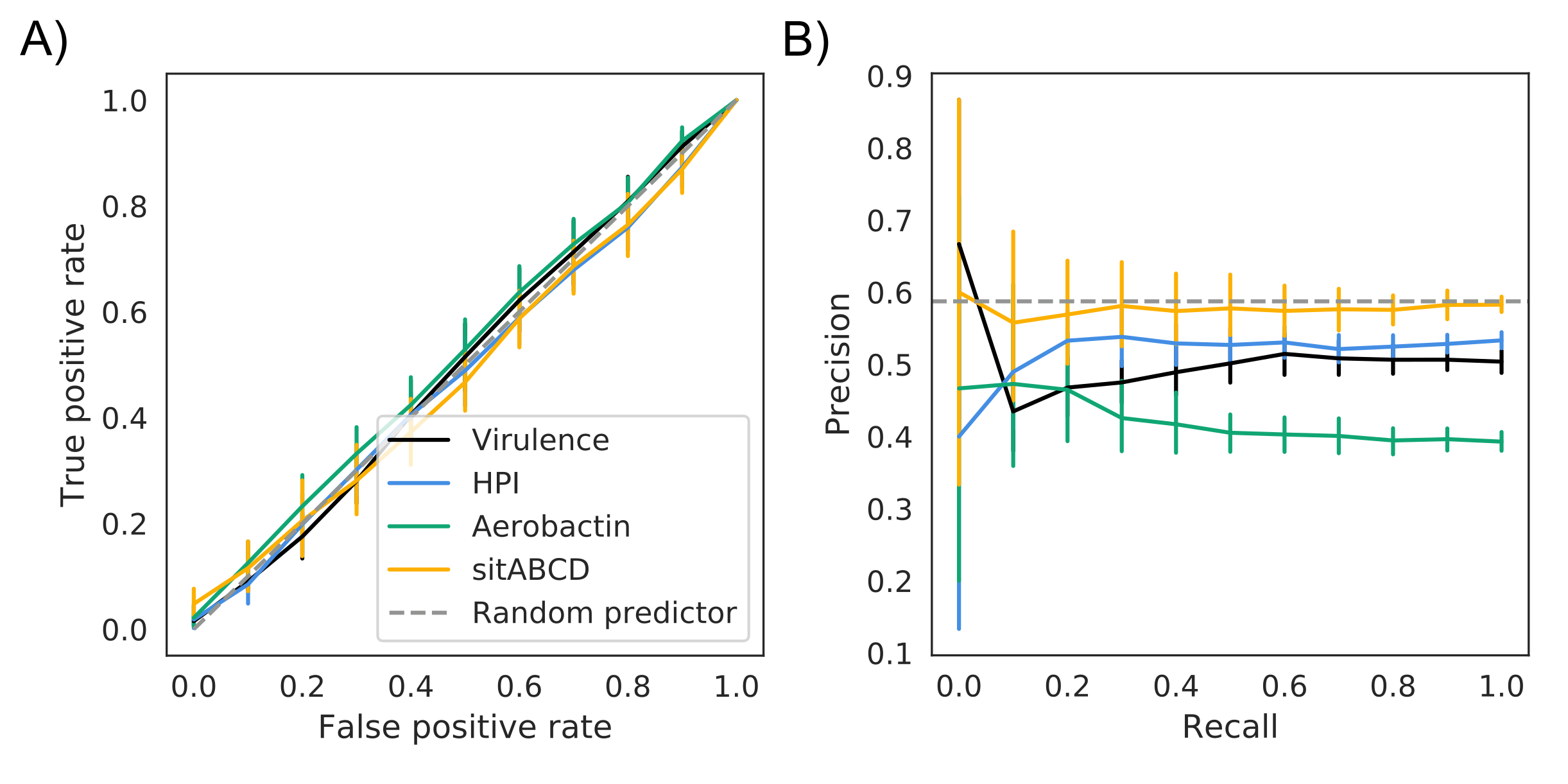

Supplement: S5 Fig — Each line except the “Random predictor” represents the mean of 15 predictors built with suffled labels for the target variable. Vertical bars represent the 95% confidence interval. (TIFF) [file pgen.1009065.s005.tiff]
