# Supplementary material for: Major role of iron uptake systems in the intrinsic extra-intestinal virulence of the genus Escherichia revealed by a genome-wide association study
Source: PLoS Genet. 2020 Oct 28;16(10):e1009065. doi: 10.1371/journal.pgen.1009065 (PMC7592755; doi:10.1371/journal.pgen.1009065)
Supplement: Attachment — Submitted filename: response_to_reviewers.docx [file pgen.1009065.s013.docx]

**Reviewer #1:**

In this manuscript, Galardini et al. describe a GWAS approach to understand the genetic underpinnings of a virulence phenotype in *E. coli*. Despite a modest sample size of genomes, they identify a top hit locus (HPI) with a large effect size, and a few other significant loci also involved in iron capture. They validate the HPI locus using knockout experiments in a mouse model of virulence, and also exploit an existing dataset of *E. coli* growth curves under different culture conditions to ask which growth conditions are correlated (or anti-correlated) with virulence, or the presence/absence of HPI or other top GWAS hits. This last analysis reveals a putative ‘collateral sensitivity’ in which virulence, HPI and other GWAS hits are anti-correlated with growth on sub-inhibitory concentrations of antibiotics in the presence of indole. Based on this result and prior experiment evidence, they conclude that iron may be deleterious to bacterial cells in the presence of antibiotics and other stressors. Thus, the presence of HPI and other iron capture systems may be necessary for virulence but deleterious in the presence of antibiotics. Overall, this study is a solid application of bacterial GWAS to an interesting phenotype (virulence in a mouse model). The synthesis with a larger phenotypic screening dataset is also appreciated, adding value to both studies. I enjoyed reading the paper, and suggest the following to improve it:

**1)** In my view, the idea of ‘collateral sensitivity’ between virulence factors and antibiotic resistance is the most valuable part of the study, and thus the most important part to get right. However, I had some trouble understanding some of these analyses (last results section) and what conclusions could be drawn. First, ‘collateral sensitivity’ is used in the abstract and throughout the manuscript, but never properly defined. The term is normally used to refer to selection for one antibiotic resistance resulting in increased sensitivity to a second antibiotic, but here it is used in a slightly different (but presumably analogous) context. It should be defined, with reference to earlier literature on this topic. Second, it is a bit unclear what can be concluded about the mechanisms of collateral sensitivity based on the phenotypic screen correlations and machine learning analyses. I appreciate that the conclusions are supported in a Discussion paragraph that brings in other experimental evidence (lines 272-291) but parts of the analyses could be better explained. For example, I also appreciated the attempt to determine what is driving the negative correlations between virulence, HPI and growth with indole + antibiotics (lines 190-203). The authors state that the Pearson’s correlation coefficients are ‘comparable’ (line 199), but it is not really clear if they are really of similar magnitude without confidence intervals. Ideally, I think that partial correlations should be computed to assess the correlation between HPI/aerobacterin/sitABCD and growth on tetracycline after controlling (or partialling out) the effect of the presence of known tetracycline resistance genes. Otherwise, the results are inconclusive.

We thank the reviewer for raising these points; indeed we also find that the “collateral sensitivity” concept we address in the discussion section is interesting and worthy of future work. In particular, the relationship between iron, indole and antibiotic efficacy might open the possibility for new therapeutic strategies. We however wish not to make extraordinary claims without sufficient evidence, and have therefore restructured the discussion about the correlations with the previously collected growth data in two sections.

The first one focuses on the positive correlations between virulence, GWAS hits and growth in the presence of antibiotics. As suggested by the reviewer we have employed partial correlation to control for the presence of resistance genes and found that the observed correlation is much weaker (Pearson’s r < 0.1) as a result of this correction. This in turn suggests that there might not be a direct causal relationship between iron homeostasis and resistance to tetracycline. The observed correlation could then be the result of the pressure to acquire antimicrobial resistance genes for pathogenic strains, as those might be more likely to encounter antimicrobials in the human host. We have therefore made this point clearer in the first part of our discussion about correlations with growth data.

In the second part we instead focus on the negative correlations, for which we introduce and discuss the idea of “collateral sensitivity” between iron, indole and antibiotics, and its proposed molecular mechanism. We unfortunately cannot apply the same approach as tetracycline to rule out an indirect relationship rather than a causal one, but regardless of whether our proposed molecular mechanism is correct or not, the negative correlation we have observed might inspire future work by us or other researchers in the community to decisively shed light on the possible “achille’s iron heel” of *Escherichia* virulent strains. As suggested we have explicitly defined the expression “collateral sensitivity” and added appropriate references.

**2)** The authors also chose to include several members of the genus in the GWAS analyses, in addition to just *E. coli*. However, it is not clear if the addition of these extra species help or hinder the analysis. From Fig S3, it seems that the long branches in the phylogeny might be adding more noise (e.g. bigger pan genome) but not adding to the association signal which seems to be coming mainly from *E. coli*. The rationale for including multiple species should at least be discussed.

We thank the reviewer for this comment; we have repeated the GWAS analysis on members of the *E. coli* species only, to verify that the long branches in the phylogeny were not adding significant noise. We report nearly identical results as shown in the figure below, in which each point represents a gene for which we report the lowest unitig p-value. We have added a short sentence explaining the rationale for including a broader phylogenetic repertoire in the results section.

**3)** The rationale for the experiments presented in the paragraph starting on line 133 is not clear. The paragraph begins by describing previous evidence that deletions in the HPI locus usually, but not always, reduce virulence. It is thus unclear why further experiments are necessary. The authors state the goal is to have a ‘broader view’ (line 139) but this is quite vague. Moreover, if the goal is to have a broader view (e.g. why is the attenuation phenotype observed when deletions are done in some lineages but not others?) this would probably require studying many more deletion mutations than just two.

We acknowledge how the addition of two deletion mutants to those thus far described in the literature is insufficient to conclusively understand the influence of different genetic backgrounds to the relationship between HPI and virulence. We have edited the paragraph to avoid stating such a claim. We however hope that the addition of two new strains including a new phylogroup (A) to those for which a HPI deletion exists is of value for the community interested in this virulence factor. Taken together with the previously described deletion mutants we believe to have convincingly demonstrated how the HPI is a causal factor of extraintestinal virulence in *E. coli* in the murine model.

**4)** Random forests analyses: To assess whether the trained models are significantly better than random, I would like to see the models trained multiple times on data with shuffled labels, to create a null distribution. This would allow to calculate a p-value for the ‘real’ model to be better (ie. better AUC or F1 score) than expected by chance. Currently, the line x=y plotted in fig 3E is not really informative. In reality, there will be a confidence interval around this line. Finally, if the aerobactin model is not reliable (as mentioned on line 209), perhaps it should be excluded from Figure 3G.

We thank the reviewer for the suggestion; it is indeed more appropriate to use label shuffling to construct an empirical random predictor to compare against. As reported in the Methods section we have trained 15 predictors after shuffling the labels of the target variable, and by keeping the same procedure for training and testing as the original predictors. As shown in the plots below (also added as Supplementary Figure 5), the original “random predictor” is within the 95% confidence interval in the ROC curve (panel A) and even higher for the precision recall curve plot (panel B). In light of these results we argue that the Aerobactin predictor should remain in the original figure, since it is still significantly better than the empirical random.

**5)** The article was generally well-written, but there are a few awkward phrases and a few non-standard idioms (e.g. the frequent use of the term ‘over’ when ‘out of’ is meant, as in a fraction or proportion). We have made an effort to find all those instances and we have corrected them extensively.

Minor/specific comments:

- line 71: A brief definition and perhaps a citation or two would be useful for ‘collateral sensitivity.’ As discussed above we have addressed this point

- paragraph starting on line 87 explaining the rationale for GWAS:

- line 89-91: large sample size will not necessarily break up the clonal frame. This depends entirely on whether additional samples bring greater phylogenetic diversity, and whether newly sampled phylogenetic groups have independent acquisitions of the genotype and phenotype of interest. We recognize that we have used confusing language here, and changed the text to indicate how a large and genetically diverse set of samples is needed to observe multiple independent acquisitions of the causal variants, therefore distinguishing them from those that define each broader lineage. This point also gives an indirect rationale for our decision to include species other than *E. coli* *sensu stricto*, which we have spelled out in the results section.

- line 94-7: unclear why the genetic determinants of virulence should have high penetrance. We have corrected our statement to indicate how at least some virulence factors should have high penetrance, given how they open up a new ecological niche and because at least some of them spread through HGT (horizontal gene transfer), potentially leading to a high prevalence across clades.

-line 36: taxons should be taxa Corrected

-line 87: unitigs should be briefly defined here We have added a brief definition the first time the term is introduced, which we hope will help readers that are unfamiliar with the term.

- line 101: its should be it is Fixed

- line 107-108: unclear and awkward phrasing. We believe we have improved this sentence indicating how the presence/absence patterns of the genes we refer to are associated with virulence and not the presence of other genetic variants such as SNPs mapping to those genes.

- line 113: how were other virulence factors defined? Presumably from the literature, but is this a comprehensive list of virulence factors? While we recognize how this list is likely not comprehensive, it is however representative of various virulence mechanisms (adhesins, toxins, protectin, iron capture systems) and it is derived from the literature. We have added a cautionary sentence to indicate how the list is not comprehensive as well as a source reference.

- line 114-115: unclear if these unitigs pass the threshold or not We realized how we might have generated some confusion with the way the reference to Supp. Figure 3 was added; in that figure we report the genes’ presence/absence pattern and not that of the unitigs. Since no unitig passing the association p-value threshold maps to the known virulence factors we have added a clarification statement.

- line 115: It would be worth connecting these 33 genes to Fig 1D, presumably the points off the diagonal, with frequency close to 1? We have made changes to Figure 1B, C and D to highlight those “core genes” (now in red), as well as adding a discussion of their function in the results section, which we believe adds some interesting points for discussion and for future experiments.

- line 133: “KO” gene should be ‘Gene knockout” Changed

- line 134: Please specify that ‘the studies’ refers to previous studies (E.g. ref 17) We have reworded the sentence slightly so that it should be clear that it is referring to ref. 17.

- line 349: presumably contigs smaller than 200bp were excluded? We thank the reviewer for spotting this typo

- line 354: It is implied that the pan genome association is between each coding gene and the phenotype, but this should be specified. Unitigs should also be defined. We have changed pangenome to “gene presence/absence patterns”, which is more explicit. Unitigs have been defined in their first appearance in the Results section.

- line 359: it is not clear if and how these p-value threshold are corrected for multiple hypothesis tests. We have added an explicit reference to multiple-testing correction

- line 366: where should be were Fixed

- lin 395: Please define s-scores. We added a short definition together with a reference.

Figure 1. In panel B, the points are sized according to the gene length fraction, but the significance of this is not made clear in the main text. I guess it shows that points with low p-values tend to be complete genes, whereas those with higher p-values tend to be gene fragments and thus possibly noise? Either this should be explained, or this detail removed from the plot. In panel C, it is unclear what distinguishes “other genes” in red from “all genes” in grey. Is it a p-value cutoff?

We agree that we did not stress the value of the “gene length fraction” metric as a way to distinguish associated genes from associated short variants (such as SNPs). We have added a short sentence in the results section and highlighted the core genes in the same way as panel 1D to better explain this point. Regarding panel C, we have overhauled the plot so that not all genes in the pangenome are represented, but only those that have unitigs mapping to them. We hope this greater consistency will reduce confusion and help the readers.

Fig S1: The pks2 gene was used as the target ’true positive,’ but it is not really clear how the precise gene identity would matter in these power simulations. Is it because the ‘real’ distribution of pks2 presence/absence across genomes was used, or simply the real fraction of genomes containing the gene? If similar results were obtained for another gene (fabG), it is not clear how this should be interpreted. Is this gene present at lower frequency? Presumably the power depends partly on the frequency, but also on the extent to which genes are distributed in different clades of the phylogeny. These power calculations are certainly worthwhile, but deserve some more explanation. We agree that this analysis could be carried out and explained better, and we thank the reviewer for pointing out these flaws. To verify how statistical power changes across different allele frequencies we picked 5 random genes in each bin in the allele frequency interval [0.1 - 0.9] and ran 20 simulations for each gene and odds ratio combination. For each gene we use the actual presence/absence patterns and we simulate the phenotype vector according to the selected odds ratio. As can be observed in the new version of Supplementary Figure 1 (reproduced below), statistical power appears to be slightly reduced at the extremes of the allele frequency interval (e.g. 0.1 and 0.9), but behaves similarly as the original analysis in the other bins. We have amended the Results and Methods section to better clarify these points.

It is also unclear if the simulation GWAS was corrected for population structure (the kinship matrix) as in the real GWAS? That is correct; we have amended the Methods section to make this more explicit

Also, “unrelated” is probably not the right term here, because all E. coli are likely related (i.e. new lineages are not being sampled in each study). Perhaps “non-overlapping” sample of E. coli or something that effect would be more accurate. We agree (we had in fact used the “non-overlapping” expression in the Results section) and we have amended the Methods section to improve clarity.

Data availability: The genomes are all available in Figshare, which is a convenient way of accessing this particular combined dataset. However, I just want to make sure that any new genomes reported are also deposited in NCBI before publication. We thank the reviewer for raising this point; we believe in having a transparent and open data process, and have submitted the new genome sequences raw reads in the European Nucleotide Archive. Supplementary Table 1 contains all accession numbers for all the genomes used in this study.

**Reviewer #2:**

General comments:

The authors report the results of a generally well-done, unbiased, WGS-based study designed to identify genetic and phenotypic correlates of lethality in a mouse sepsis model. Strengths include the comparatively large and diverse strain collection and the novel combination of WGS analysis, in vivo virulence assessments, and machine learning. Many opportunities exist to improve clarity, including regarding the rationale for some aspects of the study.

We thank the reviewer for these encouraging remarks. We hope to have improved clarity and readability by addressing these and the other reviewers’ comments.

Specific comments:

1. Lines 28-29, the phrase "which hints at collateral sensitivities associated with intrinsic virulence" is unclear and confusing, in part because the preceding phrase mentions "antimicrobials". (A similar phrase in line 71 is also confusing.) We have edited any part of the text that mentions “collateral sensitivity”, including the abstract, as well as a general overhaul to include clarity and address doubts about the correlation analysis and its discussion. We believe the readability if the manuscript has been greatly improved as a result.

2. Line 35 would be clearer if "the genus" were used instead of "it", which is ambiguous. This was fixed.

3. Lines 79-80 would be clearer if the host species for the clinical and commensal isolates were to be specified. This appears in line 306-307, but is relevant here. We have added this information in the results section.

4. Line 87, the unfamiliar (to this reviewer) technical term "unitigs" should be explained at first usage. This point was raised by another reviewer and we have defined the term at this location, where it was first defined.

5. Line 101 would be more grammatical if "if its" were to be replaced by "is", to match the "is" that appears earlier in the sentence; even better would be "instead is". This has now been fixed.

6. Line 102 seems to be missing a word, such as "analysis", after "association". We agree that the suggested edit improves readability and this has now been amended.

7. Line 103, the intended meaning of "showing" presumably is "which showed" or (as a new sentence) "This showed...". We agree with the latter suggestion and have amended the sentence accordingly.

8. Line 105, presumably "at least one" is meant, rather than "at least an". This has now been changed.

9. Lines 107 and 108, the meaning of the phrase "it's the presence of these genes to be associated with virulence" is unclear. This point was raised by another reviewer as well, and we hope to have improved clarity with the addition of further details.

10. Lines 108 and 109, this sentence refers to both genes/operons per se and their presence as being associated with virulence. Both are correct/acceptable, but the authors should go with one or the other, not use both in successive phrases. Consistent usage is needed here. This has been changed to improve readability.

11. Lines 98-118, this paragraph is very long and contains several different ideas/topics. Ideally, it would be broken into smaller, more accessible, more thematically unified units. We agree and have broken down this paragraph into three sections; the last section in particular has been expanded to expand on the characterization of core genome hits, which was suggested by reviewer #3.

12. Lines 113 and 114, it's confusing to have "known virulence factors" seemingly contrasted with the HPI and the aerobactin and sitABCD operons, since the latter also represent (or encode) known virulence factors. This point of confusion could be remedied by inserting "other" before "known" in line 113. We used the expression “some known virulence factors” to improve this sentence, which also addresses a comment from reviewer #1.

13. Line 115, "the remaining 33 genes" is confusing, because this number has not been mentioned up to now. Which 33 genes are these? We have expanded this last paragraph to better describe what these additional genes are as well as their function. We hope these overall changes address this comment.

14. Line 118, which genes are "those genes" is unclear. Same as above.

15. Fig. 1A, the lower key is labeled "Phylogroups", but the non-coli species and clades within the genus Escherichia aren't phylogroups, at least not in the usual sense of this term. Also, the red of the phylogroup ring blends with the red of the killer % ring, obscuring which is which. Furthermore, it's not immediately obvious which key applies to which ring; use of lines to connect each key with the corresponding ring would add clarity. We have edited Figure 1A to address these comments, and we believe this panel is now much easier to follow.

16. Fig. 1B and 1D, the meaning of the color code is unclear. Does the key in 1C apply here, too? That is correct; we have added a sentence in the figure legend to make this more clear.

17. Fig 1C, no need to abbreviate OG (which is confusing); it's spelled out in 1D, so that could be done also in 1C. Also, the key is unclear: it shows red for "Other genes" (not further specified", and gray for "All genes", but "All" literally would subsume all the subcategories listed above in the key, including "Other genes". We agree that the distinction between “other genes” and “all genes” is unclear. The former indicates the other associated genes that are not HPI, Aerobactin or *sitABCD*, whereas the latter indicates all other genes in the pangenome. In an effort to improve readability we have changed Figure 1C and removed all genes in the pangenome, as they were not adding much information. The improved figure reportes the same genes as panels B and D, and with a common color scheme. We hope these changes greatly improve clarity in general, and the distinction between gene presence hits from core genome ones.

18. Fig. 1D, AG is undefined. However, rather than defining it, a preferable approach would be to spell it out in the axis label. We updated the plot to spell out the meaning of AF.

19. Line 138, the term "this study" is ambiguous, since other studies were just cited; perhaps one of them is meant. Preferable wording would be "the present study". This has been changed now.

20. Line 142, ST should be defined early on, at first mention. We believe this is the first place in the manuscript where the ST acronym is first used.

21. Lines 135-143 implicitly suggest that previous experimental studies regarding the contribution of the HPI to virulence were limited to group B2 strains, and specifically to strains 536 and NU14. This, and the statement regarding achieving a broader phylogenetic assessment by studying strains from groups D (ST69) and A (ST10), overlooks a previous report of a virulence analysis, using the same mouse sepsis model, of an irp2 knockout of a strain from group D (ST69). One of the present authors coauthored that report (doi: 10.1016/j.micpath.2018.04.048.) We have addressed this comment and a similar one from another reviewer; we recognize that an HPI deletion was previously reported for phylogroup D and that we concede that more deletions across many more strains would be required to understand the influence of genetic background on the presence of HPI and virulence. We however hope that the value of these two additional deletions are beneficial for this manuscript and the community interested in the genetics of this virulence factor.

22. Line 146, no need to extend P values so many places beyond the decimal point. This implies false precision, and clutters the report without adding value. We agree and have corrected this.

23. Fig. 2A should have its axes flipped, i.e., to place strains [the independent variable] on the X-axis, and RLU [the dependent variable] on the Y-axis. However, RLU should be spelled out; there's no need to save space by abbreviating it, which interferes with comprehension. What 1e5 means is unclear. We have applied the suggested changes and agree that they make the plot easier to read. We have also fixed the x-axis (now y-axis) scale so that the RLU value can be easily read.

24. Fig. 2B would work better if it were split into two figures, one for each strain and its mutant. The current combined graph is overly busy, and has some superimpositions that obscure the lines. There's no advantage (other than saving space) to including both strains in the same graph, because the two strains are not compared with each other; instead, each is compared with its own mutant, and combining all the data in one graph obscures these key comparisons. We have split panel B in two panels, one for each strain, and we agree that it might help readability.

25. Lines 168-169, the meaning of "186 over 370" is unclear. The same comment applies in several other locations. This expression has been clarified every time it appears in the manuscript.

26. Lines 169-171, this sentence is unsatisfyingly vague, with its qualifiers "relatively high" and "in certain conditions". We have edited the sentence to more explicitly define “relatively high” and what “certain conditions”.

27. Line 176, the relevance of tobramycin is unclear. In general, more explanation is needed regarding why antibiotics and nutrient substrates were combined. As we have mentioned in the opening paragraph of this section, we are using data from a high-throughput phenotypic screen that we have previously published, which comprises more than 200 growth conditions (doi: 10.7554/eLife.31035). Many of those growth conditions are a combination of chemical and/or physical stressors, originally intended to probe the phenotypic potential of an *E. coli* strain collection. We believe that an in-depth description of the phenotypic screen would harm the readability and flow of the manuscript.

28. Line 180, to what "all growth conditions" refers is unclear, as is the intended meaning of "agree". We have edited the sentence for clarity.

29. Lines 182-203, this is a long and dense paragraph. Accessibility would be improved by breaking it into smaller, more accessible units. As a response to this comment and also to improve clarity regarding the correlations with growth data we have split this paragraph into multiple ones. We believe it is now much clearer and better presented.

30. Line 182, in this sentence it's unclear what is correlated with growth on these various antibiotic-containing media. Also, this list of agents is unnecessarily complex, making it difficult to follow. This has now been addressed

31. Lines 183 vs. 185, the implied distinction between "antibiotics" and "antimicrobial agents" is unclear. Ciprofloxacin, which strictly is regarded as an antimicrobial agent but not an antibiotic (because it's not a natural product), is classified here as an antibiotic, which is confusing. We have corrected the sentence to include all reported compounds under the more general term “antimicrobial agents”.

32. Lines 199-200, the intended meaning of the complicated phrase "...which we found to be all comparable with the direct correlation between..." is unclear. We have slightly changed the sentence to reduce confusion

33. Figure 3 would be more accessible if each subfigure included an informative title, rather than just an alphabetic label that obliges the reader to consult the legend to learn what the data represent. We agree and have amended the figure accordingly.

34. Line 233 and 287 (and elsewhere), the term "collateral sensitivities" is unclear. We have defined the term the first time it is mentioned in the main text and added a reference.

35. Line 240, a word seems to be missing at the end of this sentence, after "in vivo". We thank the reviewer for spotting this error, which has now been fixed.

36. Lined 240-242, it is unclear how the phylogenetic distribution of the HPI facilitates its detection by WGS. If the intended meaning is that WGS analysis facilitates assessment of the phylogenetic distribution of the HPI, that doesn't come across from the present wording. We have edited the sentence for clarity, hoping that it is now clear that GWAS methods in bacteria favor the discovery of genetic elements that are acquired multiple times and independently across clades.

37. Line 294, this statement seems overly broad, given that the only studied manifestation of extraintestinal virulence was lethality the mouse subcutaneous sepsis model. The findings might be specific to this model, or even this particular endpoint in this model. We have reworded the sentence to reduce the confusion this statement may generate.

38. Supplementary Figure 3 is very difficult to follow. Which rings show which variables, how the keys relate to the image, the basis for tree construction, and what point the image is intended to make are unclear. We have edited the figure to improve its readability and clarity, in a similar fashion as Figure 1A. The idea behind this figure is to show how the distribution of iron scavenging systems closely matches that of virulent strains, which is not matched as well by the collection of other known virulence factors that we had selected. We hope that with the improvements we have made its usefulness becomes apparent to the reviewer.

39. The proposed hypotheses regarding mechanisms seem somewhat "after-the-fact". The rationale (if any) for studying the particular growth conditions that were selected is unclear. In light of this and other reviewer’s comments we have revised the discussion about the results of the comparison with the high-throughput phenotypic data; in particular how the observed positive correlations suggest the pressure for virulent strains to acquire antimicrobial resistance genes and alleles. While the proposed mechanism for the observed negative correlations could not be verified in the current study, we however believe it is supported by the literature and could lead to interesting follow-up studies. Regarding the choice of growth conditions, those were originally chosen to probe the phenotypic space in a larger *E. coli* strain collection and therefore they were not optimized for this study. We however believe they allow us to pose interesting questions about the relationship between virulence, iron homeostasis and growth in the presence of antimicrobials and indole.

**Reviewer #3:**

*E. coli* is a gut commensal but also an intestinal and extra intestinal pathogen. This study by Galardini et al addresses determinants of extra intestinal virulence. First they apply a GWAS approach to find genes associated with increased virulence on a mix of 370 (326 *E. coli*) commensal, pathogenic and environmental *Escherichia* strains. They identify the HPI (high pathogenicicty island), and iron uptake genes (aerobactin, sitABC) as associated to virulence. Next they validate their in silico results by in vivo deletion of HPI. Third, they seek to associate virulence to other known phenotypes, through data analysis and machine learning approach.

The results obtained in this study are solid and convincing.

We thank the reviewer for this comment.

This study validates GWAS as a powerful and unbiased approach to study important phenotypes such as virulence. Previous works using classical approaches have already identified HPI as a virulence factor (choosing to delete potential candidates and study of the phenotype). GWAS is an unbiased method allowing for the identification of such factors and candidates. However, the finding of the HPI is not the novelty here as this island was a known virulence factor. GWAS also identified iron uptake genes and the authors also mention 33 other genes (among which mtfA). It could be interesting to comment here about these other genes as well, as those could be newly identified virulence determinants (or candidates).

We thank the reviewer for pointing out an area where we could improve the manuscript; indeed, a closer look to the hits as a whole through a GO term enrichment analysis confirmed the prevalence of iron scavenging systems, as well as a term related to protein repair. After a careful analysis of the core genome hits (genes present in more than 90% of isolates and with unitigs mapped to less than 50% of the total length of the gene), we uncovered 14 genes encoded in the vicinity of the HPI (see figure below, also added as Supplementary Figure). Those genes encode functions that are compelling for their potential relationship to virulence, such as *hprRS* (response to hydrogen peroxide) and *msrPQ* (protein repair in the periplasm after oxidative stress). Their overall presence around the HPI may mean that the unitigs are associated because their presence is correlated to that of the HPI, which we are indicating in the results section. Further experiments will be needed to verify this possibility, but in the context of this manuscript we argue that the function of these genes with respect to virulence is intriguing. We have added additional information in Supplementary Table 2 to help the reader identify those genes as well as the other core genome hits.

Page 6: In vivo deletion experiments were performed using a mouse model of sepsis, where authors have deleted irp2 and looked at attenuation of virulence. I’m assuming irp1 and irp2 belong to the HPI, no explanation is given about that. We have explicitly mentioned that both genes belong to the HPI in this section of the results to avoid confusion.

There is a mix of different phypogroups and strains, and it’s sometimes difficult to follow throughout the paper. It could help if, every time, the authors could explain why they chose a given phyogroup/strain.

For example introduction and here: Lines 134-142: “The studies on the role of the HPI in experimental virulence gave contrasting results according to the strains’ genetic background. Among B2 phylogroup strains, HPI deletion in the 536 (ST127) strain did not have any effect in the mouse model of sepsis whereas this deletion in the NU14 (ST95) strain dramatically attenuated virulence. Two strains from this study belonging to B2 phylogroup/ST141 (IAI51 and IAI52) deleted in irp1 have attenuated virulence in the same model. To have a broader view of the role of the HPI in various genetic backgrounds, we constructed irp2 deletion gene mutants in two strains of phylogroup D (NILS46) and A (NILS9) belonging to STs (Sequence Types) frequently involved in human bacteraemia (ST69 and ST10, respectively).” We thank the reviewer for pointing out ways to improve the manuscript’s readability. We have clarified why we chose a broad collection of phylogroups, clades and species in the first section of the results, and edited the section about gene knockouts to refer to exhaustively reference previous work and how the two new mutants described in this study, while providing valuable data in our opinion, are insufficient to give a definitive answer on the extent of the influence of genetic background on the relationship between HPI and virulence.

Results page 7: although this section is interesting, the link between (i) the GWAS approach to link virulence phenotype and virulence factors and (ii) high throughput study of phenotypic data to link HPI and other iron capture systems remains elusive for me. Maybe it’s just a matter of writing/explaining, but this sections appears very speculative, and the link made here between virulence and antibiotics seems indirect. Following this and the other’s reviewers’ comments we have extensively amended the results and discussion sections concerning correlations with high-throughput growth data. In particular we have broken down long paragraphs into multiple ones and improved the language used to make hypotheses. We believe that while the proposed mechanism behind the correlations remains to be further elucidated it is nonetheless interesting and supported by the literature.

Line 169: “we observed a relatively high correlation”. Relatively? This point was raised by another reviewer as well, and we agree we need to be more precise here. We have added a mention to the threshold on the correlation p-value to help the reader.

Page 7, Lines 172 to the bottom of the page: difficult to read and should be rephrased more clearly. Line 176: The authors say here that there is a correlation between growth in bipyridyl and the presence of aerobactin and cite “bipyridyl+tobramycin”, why tobramycin? We agree that this needs some clarification: the growth condition that is correlated with the presence of the aerobactin operon contains both chemicals, and have amended the sentence to make this more explicit. We however believe that this and the other correlated growth conditions (pentetic acid and pyocyanin) are a convincing confirmation of the function of the HPI and the Aerobactin and *sitABCD* operons.

Line 182: “we found strong positive correlations with growth on sub-inhibitory concentrations of several antibiotics”: positive correlation of what? We have clarified this point; we meant correlation between virulence or presence of the main GWAS hits and each growth condition.

Page 8, line 206: “We used the commonly-used random forests machine learning algorithm with appropriate partitioning of input data to tune hyperparameters and reduce overfitting”. Please explain. We have added a brief mention to the separation of input data into training and test sets and a mention to the Methods section for further details.

Page 10: lines 272 and on: very speculative and I get the impression that several things are mixed up in this paragraph:

Line 276; Sub-lethal concentrations have been shown to induce indole production but at concentrations much lower (nM to µM) than the toxic concentration of indole (e.g. 5mM). I am not sure it would be accurate to make a parallel between the lower indole concentrations upon sublethal antibiotic treatment and the toxicity and ROS production by indole at mM concentrations..

Line 280: the authors are talking about indole related toxicity. Then the sentence beginning with “This toxicity has been shown to be partly iron mediated…(ref59)”. The toxicity of what exactly? Of indole? Of the antibiotic? Which antibiotic? Ref 59 cited here is not about indole but about trimetroprime, which is not mentioned by the authors. They rather say “tobramycin is the antibiotic involved in the negative correlation”. Correlation of what? Are they talking about their own data or another paper?

In the previous paragraph, the authors rather talk about aminoglycosides, tetracycline,	rifampicin and amoxicillin, and resistance to these antibiotics in a fur mutant accumulating iron. On the other hand fur deletion also confers sensitivity to other antibiotics (which the authors do not mention but it’s also in ref 48 that is cited here). All these antibiotics have different modes of action and the effect of iron might be pleiotropic.

We thank the reviewer for these comments, which we have all addressed by restructuring the whole paragraph. More precisely, (i) we have removed the “previous paragraph” on the positive correlation because the new analysis suggested by one reviewer showed arguments for a co-selection of virulence and resistance; (ii) we added references to document that the toxic concentrations of indole at the mM magnitude can be reached in vitro and possibly in vivo and that these concentrations depend of the amount of available exogenous tryptophan; (iii) we have reworded the section to improve clarity.

Other questions:

The study includes 370 among which 326 E. coli strains from 8 phylogroups, how/why were these strains chosen? We thank the reviewer for raising this point, which was also raised by another reviewer. We have added a few brief mentions of the reason why a broad phylogenetic sample set is desirable when carrying out a bacterial GWAS analysis. Specifically the need to observe multiple independent acquisitions of the causal variants across clades in order to distinguish those from so-called “passenger mutations” or lineage-defining variants. We have added two references as well (Lees et al 2016 and Power et al 2016) for the readers who wish to read more about this.
